# RNA-binding motif proteins as context-dependent regulators of tumor–immune crosstalk, genome stability, and therapeutic vulnerabilities in cancer

**DOI:** 10.3389/fimmu.2026.1797535

**Published:** 2026-04-13

**Authors:** Jun Zhang, Yunfeng Li, Hailing Han, Bingya Zhang

**Affiliations:** 1 Department of Radiotherapy, The Second Hospital of Jilin University, Changchun, Jilin, China; 2 Department of Ultrasound Medicine, The Second Hospital of Jilin University, Changchun, Jilin, China

**Keywords:** epithelial–mesenchymal transitions, immuno-oncology, immunosuppressive microenvironment, metabolic reprogramming, RNA-binding motif

## Abstract

Cancer progression relies on dynamic post-transcriptional RNA regulation to enable phenotypic plasticity, immune evasion, and therapeutic resistance. RNA-binding motif (RBM) proteins emerge as pivotal orchestrators of these processes, modulating splicing, mRNA stability, and translation in a context-dependent manner across malignancies. This article provides a narrative mechanistic synthesis of published evidence and does not report original cohort construction or predictive-model development. Here, we review how RBM-driven RNA programs promote proliferative advantages through splicing rewiring, transcript stabilization via m6A modifications, and dual oncogenic/tumor-suppressive roles. We highlight RBM contributions to epithelial–mesenchymal transitions (EMT) and metastatic niche adaptation, including isoform-specific regulation of EMT effectors and metabolic reprogramming. Furthermore, RBMs shape tumor–immune dynamics by triggering innate immune activation through RNA misprocessing, suppressing adaptive immunity through PD-L1 upregulation, and remodeling the immunosuppressive microenvironment via cytokine and metabolic circuits. RBMs also integrate RNA processing with the choice of DNA repair pathways and genotoxic stress responses, underpinning resistance to chemotherapy and radiotherapy. Finally, pharmacological targeting of RBMs, such as RBM39 degradation via molecular glues like indisulam, exploits splicing dependencies to collapse oncogenic states and overcome resistance. These insights position RBMs as therapeutic nodes for precision immuno-oncology, with implications for biomarker-driven strategies in splicing-addicted tumors.

## 1 Introduction

Cancer progression and immune evasion are increasingly viewed as dynamic processes driven not only by genetic alterations but also by regulatory mechanisms that enable rapid adaptation to environmental, immune, and therapeutic stress (1–3). While oncogenic mutations provide the foundational driver events for malignant transformation, tumor phenotypes, progression, and immune responsiveness are predominantly governed by reversible epigenetic and transcriptional regulatory programs that facilitate plasticity and adaptation in response to microenvironmental cues (4–7). Among these regulatory mechanisms, post-transcriptional RNA regulation mediated by RNA-binding motif (RBM) proteins, such as RBM15 and RBM30, has emerged as a critical layer that enables cancer cells to rapidly fine-tune cellular states through processes like m6A modification, mRNA stability, and splicing, thereby influencing tumor-immune interactions via modulation of immune checkpoints (e.g., PD-L1), cytokine production, and the immunosuppressive tumor microenvironment (TME) (8–13).

RNA-binding proteins (RBPs) orchestrate post-transcriptional regulons through mechanisms such as mRNA stability, translation control, splicing, and granule formation, thereby shaping cellular identity, bolstering tolerance to stresses like hypoxia and oxidative damage, and facilitating phenotypic plasticity in contexts including cancer and immune responses (14–20). In cancer, dysregulated RBP activity has been linked to proliferation, invasion, and resistance to therapy (21–23). Increasing evidence further indicates that RBPs influence immune-related processes, including cytokine and chemokine signaling, antigen presentation, immune checkpoint regulation, and immune cell recruitment (24–28). However, the immunological consequences of RBP-mediated RNA regulation remain incompletely integrated across cancer types. The RBM protein family represents a structurally conserved yet functionally diverse subset of RBPs with emerging relevance to tumor immunology (29, 30). RBMs regulate multiple layers of RNA metabolism and have been implicated in cell-cycle control, apoptosis, epithelial–mesenchymal transition (EMT), metabolic adaptation, and genome maintenance (31–34). Notably, RBM expression and activity often correlate with immune-related transcriptional programs, immune cell infiltration patterns, and responses to immunotherapy (35–37). At the same time, individual RBMs can exert context-dependent and even opposing roles across tumor types, complicating their interpretation as uniformly oncogenic or tumor-suppressive regulators (38, 39).

Existing RBM literature is dispersed across tumor-specific studies of splicing, m6A biology, immune escape, genome maintenance, and therapy response, which makes cross-cutting mechanisms difficult to appreciate (8–13, 29–39). A clearer synthesis is needed to explain how these RNA-centered processes converge across cancers and why the same RBM can function as either a tumor promoter or a tumor suppressor in different settings (31–39). In this Review, we address that gap by organizing RBM biology around four mechanistic axes: isoform control, m6A-coupled RNA fate, tumor-immune crosstalk, and adaptive therapeutic vulnerability, while explicitly distinguishing direct mechanistic evidence from correlative or hypothesis-generating associations.

Unlike an original prognostic-model study, this article is a narrative review that synthesizes published experimental, translational, and clinical studies relevant to RBM proteins in tumor biology, tumor-immune crosstalk, genome stability, and therapeutic targeting. The literature cited was selected for mechanistic relevance, translational significance, and representation of both supportive and contradictory findings. Because the aim was conceptual integration rather than exhaustive evidence mapping, formal systematic-review procedures, risk-of-bias assessment, and quantitative meta-analysis were not performed.

## 2 RNA-binding motif proteins: structure, function, and implications in cancer

RBP, along with its associated RBMs, is involved in RNA processing. This process occurs post-transcriptionally. RBPs interact with single- or double-stranded RNA molecules, including mRNA (40–43). This interaction is vital for the proper functioning of the cell. According to the roles played by these proteins, RBPs can be classified into different groups, including the Hu-antigen R (HuR), heterogeneous nuclear ribonucleoprotein (hnRNP) family, arginine/serine-rich splicing factor (SRSF) protein family, RNA-binding motif (RBM) protein family, among many others (22, 31). The RBM protein family is a subset of the RBP family that shares similar structural features, including the RNA-recognition motif (RRM), RNA-binding motif (RBM), ribonucleoprotein domain (RNP), cold-shock domain (CSD), and zinc fingers (ZnFs) (22). The RRM is the major structural component of the RBM protein family, with individual RBM proteins containing one or more RRM domains. RBM3 has one RRM domain, whereas RBM19 can have up to six. The RBM protein family is classified based on the presence of the RRM domain. The RBM proteins are then assigned sequential numbers based on verification of the RRM domain. So far, more than 50 RBM proteins have been reported (22). However, not all RNA-binding proteins that contain the RRM domain are part of the RBM family. Once the exact function of the RBM protein is known, it is renamed accordingly, removing the “RBM” prefix (44).

Numerous studies have established a significant correlation of the RBM protein family with the development of cancer. Members of the RBM protein family have been shown to have aberrant expression in various types of cancer, and this has been associated with increased cell proliferation, migration, invasion, and evasion of apoptosis, thereby leading to cancer development in various types of cancer, including lung adenocarcinoma, breast cancer, and hepatocellular carcinoma (31, 45–47). For example, some members of the RBM protein family, including RBM10, have been shown to have the potential to act as tumor suppressors, thereby inhibiting cell proliferation and homologous recombination. However, this has been associated with the development of cancer, including resistance to chemotherapy with cisplatin and PARP inhibitors (48). On the other hand, RBM3 has been shown to have a positive correlation with clinical outcomes and disease-free survival in colon, breast, and ovarian cancer due to its effects on protein synthesis and apoptosis. However, this protein has also been associated with aggressive characteristics and poor prognosis in esophageal squamous cell carcinoma and some colorectal cell lines (49–54). Similarly, some members of the RBM protein family, including RBM5, have been shown to increase sensitivity to chemotherapy in lung cancer, whereas RBM38 has been shown to contribute to drug resistance in breast cancer by interacting with microRNA and altering cell cycle and apoptosis regulators (55, 56). Such inconsistencies underscore the complexity of RBM functions across cancer types, including their roles in maintaining cancer stemness, epithelial-mesenchymal transition, and immune evasion (57–59).

## 3 RBM proteins in oncogenic rna regulation: splicing rewiring, transcript stabilization, and context-dependent functions

RBM-driven oncogenesis can be understood through two recurrent regulatory layers. A splicing layer reshapes isoform output to favor survival, proliferation, and cell-state change, whereas an m6A or RNA-stability layer prolongs the expression of oncogenic transcripts, metabolic programs, and stress-adaptive signals (60–83). The balance between these layers is then filtered by lineage-specific transcriptomes and cofactors, which helps explain why RBM phenotypes diverge across tumor types. The discussion below therefore emphasizes mechanistic convergence rather than a sequential catalog of individual studies (**Table 1**).

**Table 1:** Oncogenic and tumor-suppressive roles of RBM proteins across human cancers: mechanisms, key findings, and clinical associations.

RBM protein	Cancer type	Role in cancer	Key findings/mechanism	Prognostic implication	Ref
RBM5	CRC	Oncogene	RBM5 recruits lncRNA MGC32805 → prevents apoptosis, promotes migration & 5-FU resistance; promotes ΔFAS (anti-apoptotic) isoform	Not reported	(60)
RBM25	Various cancers (focus on BCL-x splicing)	Tumor suppressor (promotes pro-apoptotic Bcl-xS)	RBM25 binds GQ-2 rG4 in BCL-x pre-mRNA → favors Bcl-xS (pro-apoptotic); G4 ligands enhance this effect	Not reported	(61)
RBM25	AML	Tumor suppressor	RBM25 knockdown promotes proliferation & decreases apoptosis; controls splicing of BCL-X & BIN1 → regulates MYC activity	Low RBM25 → high MYC activity & poor outcome	(62)
RBM25	Colon cancer	Oncogene	RBM25 overexpressed; ablation suppresses growth; regulates MNK2 splicing → promotes oncogenic MNK2b isoform	High RBM25 → poor prognosis	(63)
RBM39	HCC	Oncogene	RBM39 highly expressed; silencing inhibits proliferation, migration, invasion via integrin pathway; regulates RFX1 splicing → activates FAK/PI3K/AKT	High RBM39 → prognostic value (poor)	(64)
RBM39	Various cancers (focus on RBM39 autoregulation)	Not reported (focus on mechanism)	RBM39 autoregulates via poison exon inclusion; NMR structures of RRM1/RRM2 bound to RNA	Not reported	(65)
RBM39	Gastric cancer	Oncogene	RBM39 highly expressed; knockdown attenuates growth; regulates MRPL33 splicing → promotes oncogenic MRPL33-L isoform	High RBM39 → reduced overall survival	(66)
RBM3	HCC	Oncogene	RBM3 promotes SCD-circRNA 2 production → promotes cell proliferation	High RBM3 → short recurrence-free & overall survival	(67)
RBM3	Breast cancer	Oncogene	RBM3 upregulated; knockdown decreases proliferation & metastasis; upregulates ARPC2 via 3’UTR binding	High RBM3 → worse RFS & OS	(68)
RBM5	AML	Oncogene	RBM5 highly expressed; loss impairs leukemia maintenance; activates HOXA9 transcription	Not reported	(69)
RBM10	Breast cancer	Tumor suppressor	RBM10 decreased; depletion promotes proliferation & migration; forms complex with YBX1 & PPM1B	Low RBM10 → poorer survival	(46)
RBM15	Bladder cancer	Oncogene	RBM15 & METTL3 upregulated; knockdown attenuates proliferation, invasion, migration; enhances m6A on lncRNAs	Not reported	(70)
RBM15	COAD	Oncogene	RBM15 & TMC5 increased; RBM15 regulates m6A on TMC5 → sustains TMC5 stability	Not reported	(71)
RBM15	CC	Oncogene	RBM15 upregulated; stabilizes HEIH lncRNA via m6A → promotes stemness & EMT via miR-802/EGFR	Not reported	(73)
RBM15	OS	Oncogene	RBM15 elevated; promotes m6A on THAP9-AS1 → enhances translation & malignancy	Not reported	(74)
RBM15	CRC	Oncogene	RBM15 promotes m6A on FGD5-AS1 → enhances stability → recruits YBX1 to HOXC10 promoter	Not reported	(75)
RBM15	CC	Oncogene	RBM15 knockdown suppresses proliferation, invasion, migration & tumorigenesis; inhibits JAK-STAT pathway	Not reported	(76)
RBM15	Triple-negative breast cancer	Oncogene	RBM15 elevated in basal-like BC; regulates serine/glycine metabolism genes via m6A	High RBM15 → worse clinical outcomes	(77)
RBM15	CC	Oncogene	RBM15 upregulates OTUB2 via m6A → activates AKT/mTOR signaling	Not reported	(78)
RBM15	HCC	Oncogene	RBM15 overexpressed; promotes YES1 mRNA stability via m6A & IGF2BP1 → activates MAPK	High RBM15 → worse outcome	(79)
RBM15	NSCLC	Oncogene	Lactate induces lactylation of RBM15 (K850) → stabilizes RBM15 & enhances m6A activity	Not reported	(75)
RBM15	NSCLC	Oncogene	RBM15 overexpressed; regulates KLF1/TRIM13/ANXA8 axis via m6A & YTHDF1/YTHDF2	Not reported	(80)
RBM15	LUAD	Oncogene	RBM15 elevated; promotes LDHA mRNA stability via m6A	High RBM15 → poorer OS	(81)
RBM28	Various cancers	Oncogene	RBM28 overexpressed; interacts with p53 DNA-binding domain → inhibits p53 activity	Not reported	(82)
RBM39	CRC	Oncogene	RBM39 overexpressed; promotes proliferation, migration, invasion; inhibits apoptosis; activates NF-κB	High RBM39 → worse survival	(83)
RBM47	Medulloblastoma (MB)	Not reported (part of axis)	HDAC2 promotes RBM47 expression → increases NONO → promotes tumorigenesis	Not reported	(84)
RBM47	NPC	Oncogene	RBM47 upregulated; promotes proliferation & migration; regulates BCAT1 transcription & AS with hnRNPM	High RBM47 → poor prognosis	(85)
RBM3	Esophageal cancer	Not reported (decreased linked to aggressive features)	Decreased RBM3 linked to advanced stage & metastasis; no significant survival link	Not significantly linked to survival	(52)
RBM3	NSCLC	Tumor suppressor (high expression = better outcome)	High nuclear RBM3 → prolonged OS in adenocarcinoma; no clear link in squamous cell carcinoma	High RBM3 → improved OS in adenocarcinoma	(86)
RBM3	Epithelial ovarian cancer	Not reported (high linked to favorable features)	High RBM3 → favorable subtypes, early stage, no distant metastasis; prolonged survival (not independent)	Prolonged DFS & OS (not independent)	(54)
RBM5	Lung cancer	Tumor suppressor	Rbm5 loss-of-function → more aggressive lung cancer upon NNK exposure	Not reported	(87)
RBM5-AS1 (antisense lncRNA)	CRC	Not reported (biomarker)	RBM5-AS1 elevated in plasma & tissue; potential diagnostic biomarker	Not reported	(88)
RBM6	Laryngocarcinoma	Tumor suppressor	RBM6 downregulated; overexpression suppresses proliferation, invasion, promotes apoptosis; reduces EGFR/ERK	Not reported	(89)
RBM6	HCC	Tumor suppressor	RBM6 downregulated; overexpression attenuates viability, migration, invasion; promotes apoptosis	Not reported	(90)
RBM10	Endometrial cancer (EC)	Tumor suppressor	RBM10 KO → increased growth, migration, invasion; suppresses YAP via Hippo pathway	Not reported	(91)
RBM15	ESCC	Tumor suppressor	RBM15 upregulated; suppresses proliferation, migration, invasion	High RBM15 → better prognosis	(92)
RBM25	HCC	Oncogene	RBM25 overexpressed; promotes proliferation via AS of HDAC1, ITGB3BP, etc.	High RBM25 → shorter OS & RFS	(93)
RBM47	CRC	Tumor suppressor	RBM47-OE decreases proliferation, migration, invasion; increases apoptosis; regulates CASP3, CD44, MDM2 AS	Not reported	(94)
RBM47	ccRCC	Tumor suppressor	RBM47 downregulated; overexpression represses migration, invasion, proliferation via EMT	Low RBM47 → poor survival	(95)
RBM47	CRC	Tumor suppressor (disrupted by lncRNA)	LOC101927668 recruits hnRNPD → destabilizes RBM47 mRNA → suppresses p53/p21	High LOC101927668 (low RBM47) → poor outcomes	(96)
RBM47	HCC	Tumor suppressor	LINC00862-RBM47 positive feedback loop; both suppress HCC progression	Not reported	(97)
RBM47	HCC	Tumor suppressor	RBM47 suppresses growth & metastasis; stabilizes & transcribes UPF1	Not reported	(98)
RBM47	NSCLC	Tumor suppressor	RBM47 suppresses metastasis; stabilizes AXIN1 mRNA → inhibits Wnt/β-catenin	Not reported	(99)
RBMS3	LUAD	Tumor suppressor	RBMS3 downregulated; overexpression suppresses invasion, promotes apoptosis; positive correlation with immune infiltration	High RBMS3 → improved OS	(100)

### 3.1 Splicing rewiring as a proliferative advantage in cancer

Alternative splicing (AS) is a critical post-transcriptional regulatory step that contrasts the relatively small proteome with the immense genomic background, enabling cells to dynamically adapt and regulate protein variants in response to distinct environmental signals. Conversely, in cancer, the dysregulation of AS networks, primarily due to the inappropriate expression or dysregulated activities of splicing factors, has been characterized as the prime determinant of a selective growth advantage in cancer cells, leveraging critical oncogenic processes such as apoptosis resistance, cell cycle progression, or metabolic transformation (60, 61). Typically, this dysregulation involves the selective inclusion or exclusion of exons, leading to the predominance of cancer cells expressing a balance of variants that favor survival or proliferation, thereby maintaining the unabated growth characteristic of cancer. In this manner, splicing factors, including RBM proteins, are known to play a critical role in regulating AS variants by directly binding pre-mRNA sequences or RNA secondary structures, as well as by interacting with accessory regulatory factors, particularly to potentiate the activation of oncogenic signals (62, 63).

A paradigmatic case of splicing rewiring promoting proliferation is colorectal cancer (CRC), where the splicing factor RBM5, in concert with the lncRNA MGC32805, licenses the exclusion of exon 6 from the FAS pre-mRNA (60). In particular, the AS event leads to the repression of the pro-apoptotic membrane-bound FAS (mFAS) isoform and the induction of the anti-apoptotic soluble ΔFAS isoform, thereby resisting cell death, increasing cell migration, and conferring resistance to the chemotherapeutic 5-fluorouracil (60). From a structural perspective, the ZnF-C2H2 domain of the RBM5 protein (via the “Leu650 and Arg681” residues) interacts with the “GUACG” motif in MGC32805, thereby stabilizing the complex. At the same time, MGC32805 protects the Lys645 residue of the RBM5 protein from ubiquitination by PRPF19, thereby extending the half-life of the former. Moreover, the “His665 and Leu668” residues of the RBM5 protein specifically bind the “GAACAAA” motif located in the vicinity of the FAS exon 6, thus licensing the exon skipping event and favoring the ΔFAS/mFAS balance towards cell survival (60). This mechanism not only exemplifies how splicing factor-lncRNA partnerships rewire apoptosis but also highlights epigenetic plasticity in tumorigenesis, where neoantigen function switches from tumor suppression to oncogenesis.

By contrast, the case of RBM25 illustrates a condition in which a dual-function splicing factor can regulate either proliferation suppression or induction, depending on the context. In acute myeloid leukemia, RBM25 is a tumor suppressor that regulates AS of BCL-X and BIN1, exerting inhibitory regulatory control over MYC (62). Lower levels of RBM25 indicate increased proliferation driven by MYC, and its knockdown leads to preferential production of Bcl-xl under anti-apoptotic control, rather than Bcl-xS or BIN, which are ineffective inhibitors of MYC, thereby promoting resistance to apoptosis and accelerating the cell cycle (62). In CRC, adverse outcomes are induced by overexpressed RBM25, which acts as a promoter during oncogenic splicing of MNK2. In its regulatory role, it binds a poly-G-rich sequence within exon 14a, preventing 3’ proximal sites from being chosen and hence promoting production of the anti-proliferative MNK2b isoform (63). MNK2b is involved in the activation of pro-proliferation signal-transducing pathways. Its knockdown leads to increased production of the tumor suppressor MNK2a, hence inhibiting tumor proliferation *in vitro* and *in vivo* experiments (63). This setting-specific regulation illustrates a mechanism in which regulatory takeover among splicing regulators promotes activities within the mitogen-activated protein kinase (MAPK) pathway and, hence, a proliferative advantage by utilizing regulatory versions with specific kinase activities.

Further exemplifying the proliferative leverage of AS, the role of RBM39 comes to the fore as an oncogenic splicing factor in HCC and gastric cancer. HCC shows overexpressed RBM39 binding RFX1 precursor RNA, inducing the exclusion of exon 2 and producing an N-terminally truncated RFX1 variant devoid of its transcriptional repressive function on collagen genes, resulting in the activation of the focal adhesion kinase (FAK)/PI3K/AKT signaling pathway for increased proliferation, migration, and invasiveness (64). Autoregulation further enhances the complexity of the regulatory system, where RBM39’s tandem RRM domains autoregulates its own precursor RNA through the inclusion of a poison exon, in which RRM2 determines the 3’ splice site through N(G/U)NUUUG sequence elements and RRM3/RS elements stabilize U2 snRNP at the branch point, allowing precise control of RBM39 expression to maintain oncogenic splicing events (65). For gastric cancer, RBM39 induces alternative splicing of MRPL33 by suppressing exon 3 inclusion, thereby promoting the mitosis-stimulatory MRPL33-L isoform and augmenting mitochondrial ribosomes. RBM39 downregulation by the drug indisulam biases towards the nonfunctional MRPL33-S isoform, reducing growth (66).

Collectively, these studies reveal splicing rewiring as a dynamic oncogenic strategy, where RBM proteins hijack AS to favor isoforms that evade apoptosis (e.g., ΔFAS, Bcl-xL), activate kinases (e.g., MNK2b), or amplify integrin/PI3K signaling (e.g., truncated RFX1). Therapeutic exploitation, such as targeting RBM25 with antisense oligonucleotides or RBM39 with degraders, holds promise for reversing these proliferative advantages and restoring isoform balances in cancer.

### 3.2 RBM-dependent stabilization and translation of oncogenic transcripts

The RBM proteins promote oncogenesis by enhancing or facilitating translation initiation of target transcripts, such as mRNAs, lncRNAs, or circRNAs, through direct interactions or m6A modifications, thereby increasing mitogenic or metastatic signals in HCC, breast cancer, or CRC (67–69). These include the facilitation through interaction at the 3’ untranslated region, the use of the m6A writer or readers such as IGF2BP1 or YTHDF1/2, or through nuclear or cytoplasmic shuttling to maintain oncogenic translation of target proteins (46, 70–72). In HCC, RBM3 interacts with the SCD pre-mRNA to augment formation of the SCD-circRNA2 via back-splicing, as the circRNA is stabilized by increased mitogenic signals; RBM3 knockout leads to decreased growth *in vitro* (67). For breast cancer, RBM3 interaction with the ARPC2 3’ untranslated region leads to the stabilization of the encoded ARPC2 transcripts with increased mitogenic or migration signals, with correlations to metastases or mortality (68). In acute myeloid leukemia, RBM5 stabilizes HOXA9 through transcriptional/non-splicing mechanisms mediated by its DNA-binding domain, thereby activating HOXA9 protein; its knockout attenuates proliferation *in vivo* (69).

RBM10 in breast cancer facilitates a PPM1B-YBX1 complex, in which PPM1B dephosphorylates YBX1, thereby maintaining cytoplasmic protein levels and promoting proliferation and migration; RNAi induces nuclear phosphorylated YBX1, accelerating xenograft growth (46). RBM15, together with METTL3, m6A-modifies lncRNAs and modulates stability and translation in bladder cancer, facilitating malignancy; RNAi reduces m6A and weakens malignancy in a rat malignancy model (70). In colon adenocarcinomas, RBM15 m6A-stabilizes TMC5 mRNA and increases TMC5 protein to facilitate EMT and glycolysis and inhibit apoptosis and ferroptosis through validation of MeRIP-confirmed targets (71). RBM15 m6A-stabilizes cervical HEIH and acts as a sponge for miR-802, increasing EGFR and stemness/proliferation; additional validation involves bidirectional luciferase assays (73). In osteosarcomas, RBM15 m6A-modifies lncRNA THAP9-AS1, increasing its stability and translation, thereby enhancing viability and invasion; RNAi of RBM15 slows xenograft growth (74). In CRC, RBM15 m6A stabilizes lncRNA FGD5-AS1, recruiting YBX1 to the HOXC10 promoter to drive transcriptional activation and protein upregulation, thereby promoting proliferation (75). RBM15 knockdown in cervical cancer inhibits JAK-STAT via reduced pathway proteins, suppressing proliferation/migration (76).

In triple-negative breast cancer, RBM15 m6A modifies serine/glycine genes (PHGDH/PSAT1/PSPH/SHMT2), stabilizing their mRNAs for metabolic reprogramming and growth (77). RBM15 m6A-upregulates OTUB2 mRNA stability in cervical cancer, activating AKT/mTOR; MeRIP shows reduced m6A upon knockdown (78). In HCC, RBM15 m6A-modifies YES1 mRNA, IGF2BP1-dependently stabilizing it for MAPK activation and invasion; RNA decay assays confirm (79). In lung adenocarcinoma (LUAD), lactylation at RBM15 K850 stabilizes the protein, maintaining m6A activity/METTL3 binding to promote proliferation/migration (72). In non-small cell lung cancer (NSCLC), RBM15 m6A upregulates KLF1 via YTHDF1-stabilized mRNA while downregulating TRIM13 via YTHDF2, preventing ANXA8 ubiquitination and enhancing ANXA8 translation to promote metastasis (80). RBM15 m6A stabilizes LDHA mRNA in LUAD, elevating LDHA protein levels for glycolysis/proliferation; silencing reduces stability (81). RBM28 in various cancers translocates to the nucleoplasm upon DNA damage (Chk1/2 Phosphorylated at Ser122), binds the p53 DNA-binding domain to inhibit p53 transcription, and stabilizes anti-apoptotic states (82).

**Figure 1:**
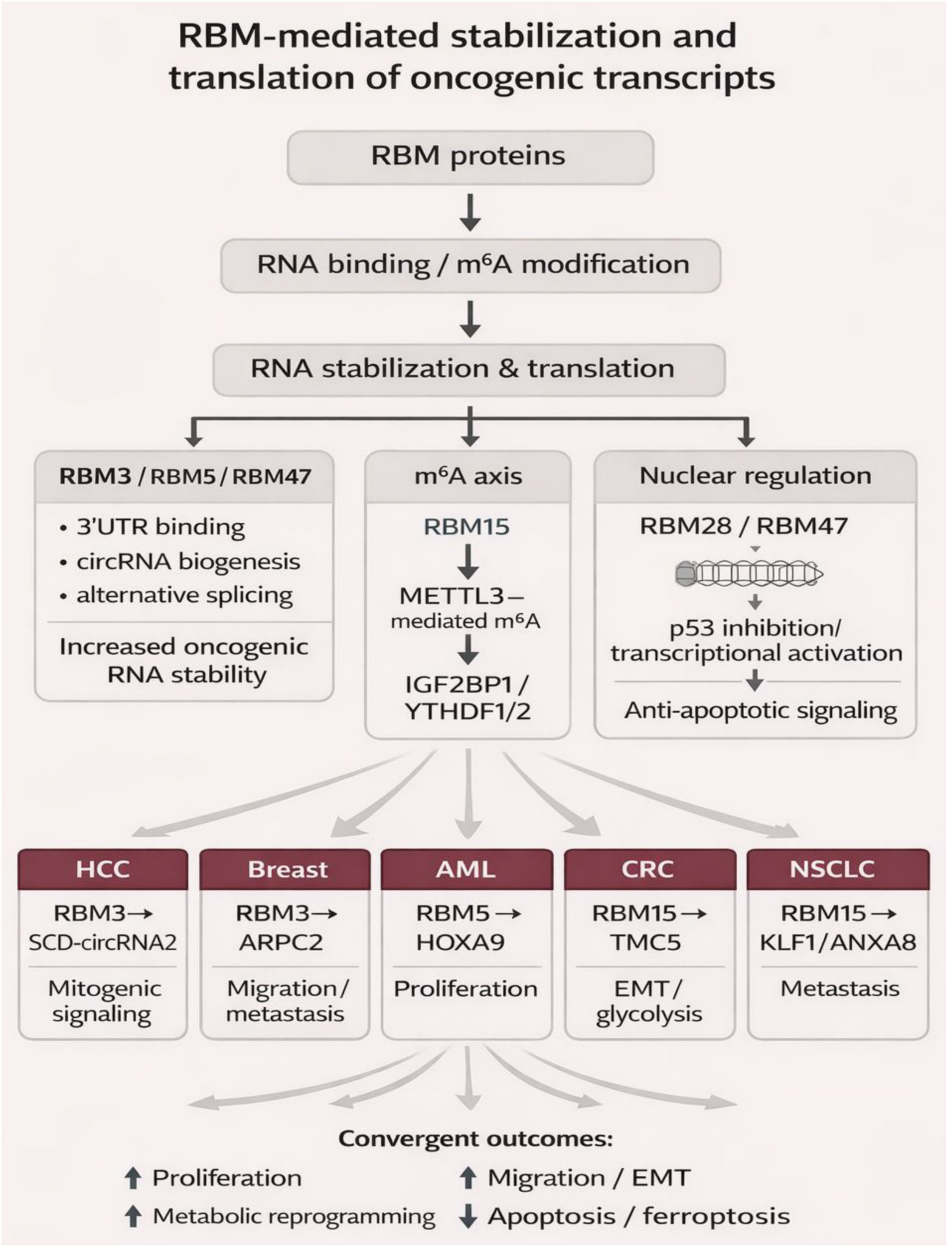
RBM-mediated stabilization and translation of oncogenic transcripts. RBM proteins promote tumorigenesis through direct RNA binding and m6A-dependent post-transcriptional regulation, leading to enhanced stability and translation of oncogenic mRNAs, lncRNAs, and circRNAs. Key mechanisms include 3′UTR interactions, circRNA biogenesis, alternative splicing, and RBM15–METTL3–m6A axis engagement with m6A readers such as IGF2BP1 and YTHDF1/2. These coordinated processes activate oncogenic signaling pathways and converge on increased proliferation, migration/EMT, metabolic reprogramming, and resistance to cell death across multiple cancer types.

RBM39 in CRC regulates the NF-κB signaling pathway to facilitate proliferation/migration/invasion and to inhibit apoptosis; RNA-seq validates pathway enrichment, and knockdown inhibits xenograft growth (83). In medulloblastoma, the HDAC2 protein catalyzes the deacetylation of H3K27, increasing transcription of the RBM47 gene and promoting NONO expression by ChIP-qPCR, which binds and stimulates proliferation/migration; knockdown increases sensitivity to temozolomide (84). In nasopharyngeal carcinoma, RBM47 binds the BCAT1 promoter to activate transcription and cooperates with hnRNPM to regulate AS of cancer-related pre-mRNAs, stabilizing oncogenic isoforms to promote proliferation/migration (85). In summary, RBM proteins coordinate oncogenic transcript stabilization via m6A modifications, RNA binding, and pathway activation to converge on sustaining tumorigenic progression in various cancers. The usage of these mechanisms emphasizes the importance of RBM proteins as critical post-transcriptional regulators, with dysregulation uncovering therapeutic opportunities. The specific RBM pathway, including RBM15/m6A or RBM47/hnRNPM, could provide the rationale for precision therapies (**Figure 1**).

Splicing-dependent and m6A-dependent RBM functions are not fully separate layers. In many settings, alternative splicing determines which transcript or isoform is available, whereas m6A deposition and reader recruitment determine the stability, localization, or translation of that RNA. Current evidence suggests that EMT, DNA repair choice, and isoform-driven signaling are more often rooted in splicing control, whereas checkpoint regulation, cytokine circuits, and metabolic immune remodeling more commonly involve m6A-coupled RNA stabilization. However, these categories are not absolute, and their hierarchy remains an open mechanistic question (60–83).

### 3.3 Context-dependent oncogenic versus tumor-suppressive RNA programs

RBM proteins, as well as their respective lncRNAs, have been shown to exhibit context-specific functions in cancers, switching between oncogenic and tumor-suppressor roles depending on tumor type, stage, or molecular interactions (52, 54, 86). Such factors have been implicated in modulating RNA stability, splicing, or pathways such as Wnt/β-Catenin or p53, leading to malignant phenotypes in cancers, including ESCC, NSCLC, and HCC (87–89). Dysregulation often correlates with clinical outcomes, highlighting its potential as a biomarker or therapeutic target (7–9). It has been reported that down-regulation of RBM3 has been correlated with aggressive factors such as late UICC stage and distant metastasis in EAC patients, and higher tumor stage/lymph node in ESCC patients, in the absence of a prognostic association (52). By contrast, in NSCLC patients with adenocarcinoma, high nuclear RBM3 protein expression has been shown to independently predict a favorable outcome in terms of overall survival and recurrence-free interval, a mechanism potentially involving RNA metabolism (86). High levels of RBM3 have been associated with favorable subtypes (mucinous/endometrioid/clear cell), early FIGO stage, and lack of distant metastasis in patients with epithelial ovarian cancer; it reinforces DFS and OS by promoting apoptosis and modulating the cell cycle (54).

As a tumor suppressor in lung cancer, RBM5 deletion in mice exposed to the tobacco carcinogen NNK accelerates adenocarcinoma, with increased nodule number and size, thereby explaining the pro-tumorigenic role of 3p21.3 deletion (87). Circulating antisense long-noncoding RNAs RBM5-AS1, VPS9D1-AS1, and STEAP3-AS1, overexpressed in CRC, serve as biomarkers with a high area under the curve value of 0.82-0.94, suggesting the activation of Wnt/β-catenin (88). RBM6 suppresses the progression of laryngocarcinoma and HCC by inhibiting proliferation/migration/invasion, as well as EGFR/ERK signaling, resulting in increased cleaved caspase-3 (89, 90). RBM10 inhibits proliferation/migration/invasion in endometrial cancer by increasing YAP phosphorylation and suppressing the Hippo-YAP pathway, as shown by RNA-seq and KEGG analyses (91). High expression of RBM15 in ESCC is associated with improved survival with the suppression of cell proliferation/migration/invasion (92). On the contrary, the overexpression of RBM25 in HCC is a predictor of poor survival, particularly in higher stages and with microvascular invasion (MVI), suggesting that RBM25 positively regulates proliferation via alternative splicing of AS cell-cycle/division-related genes, such as HDAC1/ITGB3BP (93). RBM47 exerts tumor-suppressive effects in CRC by regulating AS and expression of proliferation/apoptosis genes (CASP3/CCN1/ATF5/CD44/MDM2), inhibiting invasion/migration (94). In renal cell carcinoma, RBM47 downregulation promotes EMT by modulating RNA stability, correlating with poor survival (95).

LncRNA LOC101927668, which is amplified in CRC, interacts with hnRNPD to destabilize RBM47 mRNA through AU-rich elements (AREs) within the 3′UTR, inhibiting p53/p21 signaling and progressing to enhanced proliferation/metastasis (96). In HCC, LINC00862 forms a positive feedback loop with RBM47, recruiting CHD5 to the RBM47 promoter, thereby increasing their expression and suppressing tumor growth/invadopodia-based invasion (97). RBM47 promotes the transcription of UPF1 mRNA as an SSB-DNA-binding protein, enhancing nonsense-mediated decay (NMD) and inhibiting HCC progression (98). NSCLC’s RBM47 binds to and stabilizes AXIN1 mRNA, thereby inhibiting Wnt/β-catenin signaling and perturbing proliferation/invasion (99). RBMS3 in LUAD suppresses invasion/apoptosis while accelerating G1-S transition, correlating with immune infiltration (B/CD8+/CD4+ T cells/macrophages/neutrophils/dendritic cells) (100).

**Figure 2:**
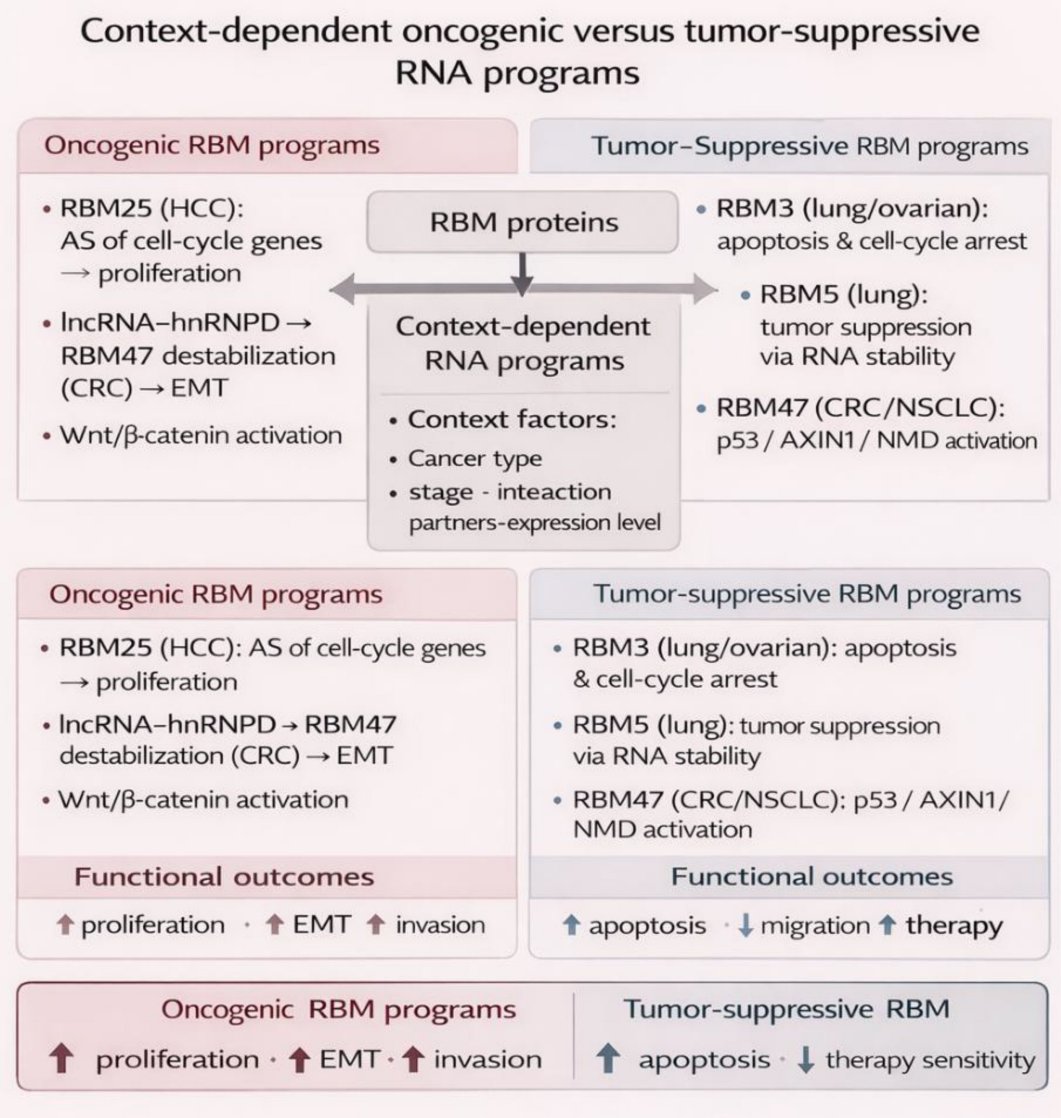
Context-dependent oncogenic versus tumor-suppressive RNA programs mediated by RBM proteins. RBM proteins exhibit dual functions in cancer by orchestrating RNA stability, alternative splicing, and pathway regulation in a context-dependent manner. Depending on cancer type, stage, interaction partners, and expression levels, RBMs can drive oncogenic programs (e.g., RBM25-mediated splicing and Wnt/β-catenin activation) or tumor-suppressive programs (e.g., RBM3/RBM5/RBM47 via apoptosis, p53, AXIN1, and NMD pathways). These opposing RNA programs result in divergent functional outcomes, including enhanced proliferation and EMT or increased apoptosis and therapeutic sensitivity.

In summary, context dependency in RBM biology should be interpreted mechanistically rather than descriptively. The phenotypic direction of a given RBM is likely shaped by four determinants: the lineage-specific transcript pool available for binding, the surrounding chromatin and epitranscriptomic state, the set of interacting RBPs or lncRNAs, and selective pressures imposed by metabolism and the immune microenvironment (38, 52, 54, 86–100). Under one combination, RBMs reinforce apoptosis, nonsense-mediated decay, or Wnt restraint; under another, they favor oncogenic splicing, survival signaling, and invasive plasticity. This framework helps explain why RBM expression alone is an incomplete predictor of biological outcome and why cofactor usage and cellular context must be considered when translating RBM biology into therapy (**Figure 2**).

## 4 RBM proteins in epithelial–mesenchymal transitions and metastatic progression: splicing-driven plasticity and niche adaptation

**Figure 3:**
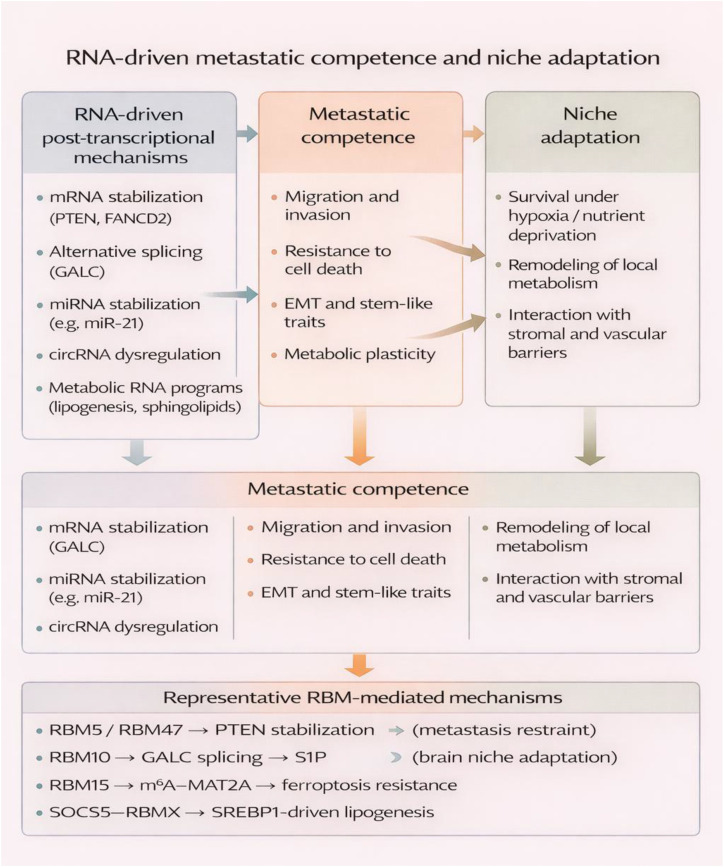
RNA-driven metastatic competence and niche adaptation mediated by RBM proteins. RNA-binding proteins orchestrate post-transcriptional regulatory programs, including mRNA stabilization, alternative splicing, noncoding RNA regulation, and metabolic RNA networks. These RNA-driven mechanisms endow cancer cells with metastatic competence, characterized by enhanced migration, invasion, survival, EMT, and metabolic plasticity. Subsequent niche adaptation enables successful colonization of distant microenvironments through metabolic remodeling, stress tolerance, and interactions with stromal and vascular barriers.

RBM-dependent control of EMT and metastasis can be organized into three recurring processes: isoform switching that changes migratory signaling, loss of epithelial restraint that increases plasticity, and RNA-mediated adaptation to distant metastatic niches. The discussion below focuses on these convergent mechanisms rather than exhaustive study-by-study description (**Figure 3**, **Table 2**).

**Table 2:** Context-dependent roles of RBM proteins in promoting or suppressing EMT and metastasis through post-transcriptional mechanisms.

RBM protein	Cancer type	Expression	Role	Mechanism	Functional effects	Ref
RBM39	Colon cancer	Not reported	Oncogene	MORC2 binds RBM39 → CDK5RAP2 splicing switch (L to S) → CDK5RAP2 S recruits PHD finger protein 8 → promotes Slug transcription	Promotes EMT, invasion, metastasis	(101)
RBM10	NSCLC	Low	Tumor suppressor	Binds Neat1 → regulates AS (promotes Neat1_1, inhibits Neat1_2) → upregulates PTEN → inhibits PI3K/AKT/mTOR	Inhibits invasion, metastasis	(102)
RBM10	CRC	Decreased	Tumor suppressor	Interacts with SNHG17 → regulates splice isoforms (inhibits SNHG17_2)	Inhibits invasion, EMT	(103)
RBM47	Breast cancer	Not reported	Tumor suppressor	Binds mRNAs (introns/3’UTRs) → alters splicing/abundance (e.g., stabilizes DKK1 → inhibits WNT)	Inhibits re-initiation, growth, progression, metastasis	(104)
RBM10	Thyroid cancer	Loss	Tumor suppressor	Loss → exon inclusion in VCL, TNC, CD44 → increases RAC1-GTP	Promotes metastases, invasiveness	(105)
RBM47	CRC	Downregulated	Tumor suppressor	Activated by FOXA1 → silenced by CpG methylation → induces MET	Inhibits migration, invasion, EMT	(38)
RBM5	CRC	Downregulated	Tumor suppressor	Stabilizes PTEN mRNA → inhibits PI3K/AKT	Represses proliferation, migration, invasion, glycolysis	(106)
RBM10	LUAD	Downregulated	Tumor suppressor	miR-224-5p targets RBM10 → inhibits p53; p53 affects RBM10 via miR-224-5p	Inhibits proliferation, metastasis	(107)
RBM6	Prostate cancer	Not reported	Oncogene	Suppresses CDH1; under high ZEB1 → enhances MMP16 inhibition	Promotes migration (normal); inhibits under high ZEB1	(108)
RBM10	HCC	Upregulated	Oncogene	Stabilizes miR-21	Promotes proliferation, migration, invasion	(109)
RBM15	OS	Upregulated	Oncogene	m6A on MAT2A → promotes expression	Enhances proliferation, migration, invasion; inhibits ferroptosis	(110)
RBM10	LUAD	Deficiency	Tumor suppressor	Deficiency → GALC splicing inhibition → upregulates GALC & S1P → increases BBB permeability	Promotes brain metastasis	(111)
RBM39	ESCA	Upregulated	Oncogene	Binds FANCD2 3’UTR → stabilizes mRNA	Promotes proliferation, migration, invasion, metastasis	(39)
RBM47	CRC	Not reported	Tumor suppressor	SPR via miR-181c/d-5p targets RBM47 → inactivates PI3K/AKT	Suppresses proliferation, invasion, migration	(112)
RBM47	OS	Not reported	Oncogene	Binds flanking introns → facilitates circFNDC3B → reduces FNDC3B mRNA; IGF2BP1 binds circFNDC3B	Promotes malignant processes	(113)
RBM47	HCC	Not reported	Tumor suppressor	miR-122 suppresses RBM47 → downregulates ITGAV → activates TGF-β	Inhibits metastasis, migration, invasion	(114)
RBMX	HCC	Not reported	Oncogene	SOCS5 binds RBMX → costimulates SREBP1 promoter → induces lipogenesis	Promotes metastasis	(115)

### 4.1 Alternative splicing as a molecular engine of cell-state transitions

AS is an essential regulatory step in cell-state transitions, including EMT, and mediates cancer cell invasion and metastasis. In CRC, Microrchidia family CW-type zinc finger 2 (MORC2) interacts with the RBM39, where MORC2 binds to the RRM1 domain of RBM39, whereas RBM39 binds to site 1 of the pre-cyclin-dependent kinase 5 regulatory subunit-associated protein 2 (CDK5RAP2) exon 32, which involves interaction of the UHM domain of RBM39 (101). This interaction mediates a splicing shift from the long transcript CDK5RAP2 L to the short transcript CDK5RAP2 S, which generally stimulates EMT, cell invasion *in vitro*, and metastasis *in vivo* (101). The underlying mechanism involves CDK5RAP2 S recruiting PHD finger protein 8 to the Slug promoter, where it removes repressive histone marks, thereby inducing enhanced Slug expression and ensuing EMT (101). More importantly, CDK5RAP2 S, but not CDK5RAP2 L, is absolutely required in the induction of EMT by MORC2 or RBM39, implying the isoform requirement of CDK5RAP2 S in cell state transitions (101). High expression of MORC2, RBM39, or Slug is associated with enhanced metastasis and a poor prognosis in CRC patients (101).

In NSCLC, RBM10, with downregulated expression in cancer tissues and cells, binds multiple regions of the long non-coding RNA nuclear-enriched abundant transcript 1 (lncRNA Neat1) via crosslinking-immunoprecipitation followed by high-throughput sequencing analysis, and this binding is verified by RNA immunoprecipitation (102). RNA binding mediates AS of Neat1, producing variants Neat1_1 and Neat1_2; concomitant with RBM10 overexpression, the total level of Neat1 and the amount of variant Neat1_2 were suppressed, while variant Neat1_1 was induced; loss of RBM10 induced the reverse (102). The pro-invasive and metastatic phenotype of NSCLC is mainly mediated by variant Neat1_2, as indicated by transwell invasion and scratch test results. In the latter test, RBM10 activity suppressed the process, with concomitant reductions in variant Neat1_2; the results indicate RBM10’s suppressive function via downregulation of variant Neat1_2 (102). At the molecular level, the activity of the overexpressed RBM10 induced an increase in the expression of phosphatase and tensin homolog (PTEN). At the same time, the phosphatidylinositol 3-kinase (PI3K), protein kinase B(AKT), and mammalian target of rapamycin(mTOR) were suppressed. Consequently, the PI3K/AKT/mTOR signaling cascade sustains cell-state transitions toward invasiveness (102). This negative correlation between RBM10 and Neat1_2 underscores how AS imbalance can perpetuate metastatic potential (102).

Beyond its role in CRC, reduced RBM10 expression in tumor tissues and cells also affects the AS of the lncRNA small nucleolar RNA host gene 17 (SNHG17), as revealed by crosslinking-immunoprecipitation sequencing and RNA immunoprecipitation analysis (103). Overexpression of RBM10 inhibits CRC cell invasion in transwell assays but does not affect the protein levels of EMT molecules, whereas its knockdown promotes invasion (103). RBM10 co-regulates the balance of its splice variants with the aid of SNHG17, of which the spliced variant SNHG17_2 is increased in CRC and correlates with the promotion of invasion (103). Therefore, RBM10 inhibits the invasion of CRC by carefully regulating the AS of SNHG17, thereby preventing the shift in splice variants toward the mesenchymal form (103).

Collectively, these results reveal the complexities of AS as an active molecular machine that drives transitions of cell states in cancer, where splicing factors such as MORC2-RBM39 and RBM10 have isoform-determined functions to regulate outcomes that either activate (e.g., CDK5RAP2 S-induced Slug expression) or suppress (e.g., Neat1_2 or SNHG17_2 signaling-mediated suppression of EMT and metastasis) EMT and metastasis. The implications of these isoform imbalances, such as the predominance of CDK5RAP2 S versus Neat1_2/SNHG17_2, extend beyond the reprogramming of the transcriptome or signaling pathways, based on histone modifications or PTEN/PI3K/AKT/mTOR pathway deregulations, to link to the path of enhanced aggression in CRC and NSCLC.

### 4.2 RBMs as regulators of epithelial–mesenchymal plasticity rather than fixed EMT states

RBM47 and RBM10 are better viewed as regulators of epithelial–mesenchymal plasticity rather than fixed EMT states. By controlling RNA stability and alternative splicing, they help maintain phenotypic balance in cancer cells. Their loss does not simply produce a stable mesenchymal state. Instead, it promotes reversible plasticity, allowing tumor cells to shift between epithelial and mesenchymal programs in response to metastatic and microenvironmental pressures (38, 104, 105).

In breast cancer, RBM47 acts as a metastasis suppressor by preserving epithelial traits through post-transcriptional regulation of target mRNAs (104). Clinical and transcriptomic studies support its role in limiting tumor progression and metastatic outgrowth (104). HITS-CLIP data showed that RBM47 binds introns and untranslated regions, where it regulates splicing and stabilizes dickkopf WNT signaling pathway inhibitor 1 (DKK1) mRNA (104). Through this mechanism, RBM47 restrains WNT signaling and suppresses EMT-associated changes, whereas its loss favors adaptive traits linked to metastatic competence (104).

In thyroid cancer, RBM10 loss promotes mesenchymal plasticity by altering splicing of cytoskeletal and extracellular matrix-related transcripts (105). Loss-of-function mutations are enriched in metastatic disease, and RBM10 deficiency is associated with RHO and RAC migratory signaling (105). RBM10 deletion increases exon inclusion in vinculin (VCL), tenascin C (TNC), and CD44, with higher RAC1-GTP levels, increased migration, and greater invasiveness (105). In mouse HrasG12V/Rbm10KO models, metastatic lesions develop but are reversed by RBM10 re-expression or deletion of RBM10-dependent inclusion isoforms (105). These findings suggest that RBM10 loss supports a reversible extracellular matrix-cytoskeletal transition rather than an irreversible EMT state. This transition is also linked to NFκB pathway dependence in CRISPR-based screens (105).

In CRC, RBM47 repression drives EMT-linked plasticity through transcriptional and epigenetic mechanisms (38). RBM47 expression declines in aggressive disease and is associated with poor prognosis, particularly in CMS4 and CRIS-B metastatic subtypes (38). FOXA1 directly activates RBM47, whereas FOXA1 loss reduces RBM47 expression and is followed by CpG promoter methylation in mesenchymal-like transformed tissues and liver metastases (38). In these settings, RBM47 is required for FOXA1-driven MET and for suppression of invasion and migration (38). Thus, RBM47 silencing supports reversible EMT, invasion, and later epithelial reversion, while promoter methylation may serve as a metastatic biomarker (38).

Overall, RBM47 and RBM10 regulate the capacity of tumor cells to move between epithelial and mesenchymal states. Their inactivation promotes metastatic evolution through transient and adaptable phenotypes rather than rigid EMT states, highlighting splice-dependent isoforms, NFκB-linked dependencies, and epigenetic mechanisms as potential therapeutic targets.

### 4.3 RNA-driven metastatic competence and niche adaptation

Metastatic potential in cancer involves not only cellular reprogramming in malignancy models but also adaptation to distant environments, which requires cellular changes and the action of RBPs that precisely target post-transcriptional regulation. These models of RNA-related functions, such as mRNA stabilization, splicing regulation, and interactions with ncRNAs, have enabled cancer cells to develop the ability to migrate, resist cell death, and adapt to the microenvironment to colonize new regions. Such action has been shown in models involving the RBM family of RBPs, particularly where their dysfunction has increased metastatic potential in malignancy models of CRC, LUAD, HCC, OS, prostate cancer, and ESCA. In CRC, RBM5 functions as a metastasis suppressor by stabilizing the phosphatase and tensin homolog (PTEN) mRNA, thereby opposing metastatic properties. The results showed downregulation of RBM5 protein in CRC tissues and cells, and forced overexpression suppressed proliferation, migration/invasion, and glycolysis (reduced glucose uptake, lactate, and ATP secretion) (106). Downregulation was rescued further by PTEN siRNA, suggesting the therapeutic potential of RBM5 as an adaptation barrier that restricts niche adaptation through metabolic inhibition (106). Conversely, saponins extracted from Platycodi radix (SPR) antagonize proliferation, invasion, and migration by dephosphorylating PI3K/AKT via the miR-181c/d-5p and RBM47 pathway in CRC (112). These findings demonstrated that conditionally elevated miR-181c/d-5p expression promoted proliferation, invasion, and migration by repressing RBM47; this stabilized PTEN mRNA; and SPR treatment rescued RBM47 levels, thereby stabilizing PTEN and blocking AKT phosphorylation, hindering niche adaptation and overcoming metastatic competence in hypoxic or nutrient-poor microenvironments (112).

RBM10 deletion promotes metastatic niche adaptation, especially brain metastasis, by regulating sphingolipid metabolism. Western blot and luciferase reporter analysis confirmed RBM10 reduction (through binding of miR-224-5P to its 3’ UTR) to encourage proliferation/metastasis through a feedback loop that represses p53 (107). In brain metastatic disease, RBM10 deletion triggers exon-skipping mutations within the GALC primary transcript of galactosylceramidase (GALC), resulting in its overexpression and sphingosine-1-phosphate (S1P) production, leading to blood-brain barrier BBB breakdown and niche colonization (111).*In vitro* BBB and xenografting model validation indicated that RBM10 knockdown increases the transmigration of RBM3 cells, and this effect is reversed by Fingolimod or RBM10 chaperone rescue with its reversor drug, RU-SKI-43 (111).

RBM10 and RBM47 are oncogenic in metastasis by stabilizing miRNAs for the HCC signaling circuitry. RBM10 knockdown suppresses proliferation/migration/invasion of SNU-398 by stabilizing miR-21, while its overexpression in HepG2 augments these by stabilizing miR-21 (109). This drives the subsequent activation of the TGF-β signaling network, promoting the invasiveness of the stem cell niche. Likewise, miR-122 induction potentiates the suppression of RBM47, thereby attenuating integrin alpha V (ITGAV) mRNA stability (via RBM47-AU-rich element interaction), leading to activation of the latent form of TGF-β and enhanced motility/migration (migration assays, *in vivo* pulmonary metastasis) (114). In the steatotic subtype of HCC among HBV-cirrhotic patients, the SOCS5-RBMX complex promotes lipogenic signaling mediated by SREBP1, thereby promoting metastasis (115). Proteomics and metabolomics revealed SOCS5-SH2 domain (Y413/D443) binding to RBMX-RRM, co-activating SREBP1 promoters (co-IP, GST pull-down), increasing fatty acid synthesis to fuel membrane remodeling and niche adaptation in lipid-rich environments; high SOCS5 predicts larger tumors and worse prognosis (115).

In OS, RBM15 and RBM47/IGF2BP1 regulate metastatic ability via m6A and circular RNA dysregulation. RBM15 overexpresses MAT2A via m6A modification, stimulating proliferation and metastasis while inhibiting ferroptosis (GSH/ROS/Fe2+), thereby facilitating survival in iron-laden bone microenvironments; RBM15 knockdown repressed xenograft tumor development (110). At the same time, RBM47 was shown to bind intronic regions to form circular RNA FNDC3B (circFNDC3B), inhibiting linear FNDC3B mRNA expression and proliferation/migration, whereas IGF2BP1 competitively degraded FNDC3B mRNA, leading to a paradoxical increase in the metastatic phenotype *in vivo* (113). In prostate cancer/ESCA, context-dependent roles for RBM reflect adaptive plasticity/evolution. RBM6 inhibits migration during EMT by suppressing CDH1, but high ZEB1 overrides this, enhancing MMP16 inhibition and reducing invasion (108). In ESCA, RBM39 stabilizes the FANCD2 3’UTR, activating Fanconi anemia repair for proliferation/metastasis; knockdown impairs xenograft growth and lung metastasis, and rescue is achieved by FANCD2 overexpression (39).

RBM-controlled metastasis is not limited to local EMT. It also involves stabilization of pro-metastatic transcripts, rewiring of lipid or sphingolipid metabolism, and survival programs that support colonization of distant sites. These processes are clinically important because they also condition how tumors respond to stress, therapy, and, in some settings, immune pressure (106–115).

## 5 RBM proteins in immune modulation: innate activation triggers, adaptive evasion strategies, and tumor microenvironment remodeling

Immune effects of RBMs can be grouped into three functional layers: innate immune sensing, adaptive immune evasion, and cytokine or metabolic remodeling of the TME. The emphasis below is on immune phenotypes with functional support, while also distinguishing them from claims based only on pathway enrichment or checkpoint expression (**Table 3**).

**Table 3:** Immunomodulatory roles of RBM proteins in cancer and inflammation: mechanisms of immune regulation, evasion, and therapeutic implications.

RBM protein	Disease/cancer type	Expression/alteration	Role in disease/immunity	Key mechanism/findings	Immune effects	Ref
RBM3	Lung inflammation (asthma)	High in ILCs; induced by TSLP/IL-33	Suppressor of ILC activation	Suppresses ILC responses; ILC-intrinsic function; regulates type 2/17 cytokines & CysLT1R	Enhanced eosinophilic inflammation & ILC activation in KO; dependent on CysLT1R	(116)
RBM39	Neuroblastoma	Not reported	Degrader invigorates immunity	Induces inflammatory TME; enhances NK activity; induces cell state switch & epigenetic reprogramming	Enhances anticancer activity of NK cells; combination with anti-GD2 eradicates tumors	(29)
RBM3	Nasopharyngeal carcinoma (NPC)	High in immune resistant	Induces immune resistance	Modulates TME via cytokines (IL-2/IL-10); recruits Treg & M2 macrophages; activates p-AKT/MEK1 → IL-10	Increases Treg & M2 in TME; immune resistant TME upregulates RBM3 in tumor cells	(117)
RBM10	Lung adenocarcinoma (LUAD)	Deficiency	Increases immune activity	Higher TMB, HLA expression, immune infiltration (dendritic cells, macrophages, neutrophils, CD8+ T cells)	Higher checkpoint molecules (PD-L1, TIM-3); better immunotherapy response (higher IFNG/MSI/CD8, lower MDSC/M2)	(37)
RBM12	Hepatocellular carcinoma (HCC)	Upregulated	Drives immune evasion	Binds JAK1 mRNA via 4th-RRM; recruits EIF4A2 via 2nd-RRM → enhances JAK1 translation → JAK1/STAT1 → PD-L1	Upregulates PD-L1; facilitates immune evasion	(118)
RBM10	EGFR mutant LUAD	Not reported	Poor prognostic if high with PD-L1	High RBM10 → better OS; PD-L1 positivity → poor OS; influences EGFR-TKI PFS	PD-L1 positivity reduces EGFR-TKI PFS; high RBM10 improves PFS in Ex21	(119)
RBM10	Pancreatic cancer	Low in tumor	Inhibits development & immune escape	Suppresses PD-1 in NK via JAK-STAT; knockdown enhances proliferation/migration	Reduces NK cytotoxicity; AZD1480 restores NK activity	(120)
RBM15	Breast cancer	High	Drives progression & immune escape	Stabilizes KPNA2 mRNA via m6A → enhances proliferation/migration/invasion & suppresses CD8+ T proliferation/apoptosis	Suppresses lymphocyte immunity; blocks tumor growth *in vivo*	(121)
RBM30	Hepatocellular carcinoma (HCC)	High in tumor	Drives immune evasion	Binds DNA near STAT1 TSS; recruits DOT1L → H3K79me3 → upregulates STAT1 transcription → activates PD-L1 transcription	Enhances PD-L1 → immune evasion by binding PD-1 on T cells	(9)
RBM47	Gliomas	High in gliomas	Immunotherapeutic target & biomarker	Correlates with grade; enriched in CD163+ macrophages	Promotes macrophage infiltration	(122)
IGF2BP2	Colorectal cancer (CRC)	Higher in mCRC; increased in primary	Promotes angiogenesis & progression	Binds m6A on CEMIP mRNA → stability → CEMIP promotes growth/invasion/angiogenesis via GRP78/PI3K-AKT	Not reported	(123)
RBM15	Esophageal squamous cell carcinoma (ESCC)	Not reported	Boosts immune response via PLOD3	Regulates PLOD3; increases CD4+ T cell infiltration	Increases CD4+ T cell infiltration	(124)
RBM15	Triple-negative breast cancer	Not reported	Enhances PTX resistance	Targets m6A TNFSF9 → M2 polarization	Induces M2 polarization; decreases IL-1β/TNF-α, increases IL-10/TGF-β	(125)
RBM15	Colon adenocarcinoma (COAD)	Not reported	Aggravates progression by remodeling TME	Increases ITGBL1 mRNA stability via m6A → proliferation/migration/invasion/M2 polarization/lymphocyte immunity	Hinders lymphocyte immunity; promotes M2 polarization	(126)
RBM15	Clear cell renal cell carcinoma (ccRCC)	Not reported	Promotes growth/metastasis/macrophage infiltration	EP300/CBP acetylates promoter → upregulates RBM15 → m6A CXCL11 stability → macrophage infiltration/M2 polarization	Promotes macrophage infiltration & M2 polarization	(127)
RBM15	Pancreatic cancer	Not reported	Boosts immune response	Loss of RBM15 → increased FH → decreased fumarate → enhanced CD8+ T infiltration/activation	Enhances CD8+ T infiltration/activation	(128)
RBM15	Non-small-cell lung cancer	Not reported	Boosts radioresistance	Induces CBR3-AS1 m6A-IGF2BP3 → miR-409-3p/CXCL1 → MDSC recruitment → inhibits T cell	Limits MDSC recruitment; enhances T cell activity (when inhibited)	(129)
RBM15	Colorectal cancer	High	Boosts immune response	Loss of RBM15 → increased FH → decreased fumarate → enhanced CD8+ T infiltration/activation	Enhances CD8+ T infiltration/activation	(34)
LINC01615 (via RBMX)	Colorectal cancer	Not reported	Induces M2 polarization	Exosomal LINC01615 → scaffold RBMX-EZH2 → EZH2 expression → M2 polarization	Induces M2 polarization of TAMs	(130)
RBM3	Prostate cancer	Increased in macrophages & PCa cells	Increases in cross-talk	Cross-talk with TAMs; conditioned media induces RBM3; M1 via CXCL10, M2 via IL4/IL10	Positive correlation with macrophage infiltration	(131)
RBM25	Rheumatoid arthritis (RA)	Decreased	Restrains inflammation	Mediates ACLY splicing (L to S) → metabolism rewiring; deficiency → proinflammatory via H3K9ac/H3K27ac/HIF-1α	Increases proinflammatory mediators; spontaneous arthritis in KO	(132)
RBM47	Intestinal injury/tumorigenesis	Decreased in CRC	Modifier of pathways	KO → increased proliferation; upregulates antioxidant/Wnt/stem; protects colitis	Protects against colitis-associated cancer; promotes polyposis in aged	(133)
RBM47	Non-small cell lung cancer (NSCLC)	Downregulated in CSCs	Regulates CSC & antitumor activity	KT2440 induces m6A → upregulates RBM47 → impairs self-renewal/tumorigenesis via Wnt; destabilizes PD-L1 mRNA	Enhances T-cell proliferation/cytotoxicity	(134)
RBM15	Lung adenocarcinoma (LUAD)	Upregulated	Prognostic biomarker	Co-expressed genes related to cell cycle/M phase; correlates with immune infiltration	Correlates with immune infiltration	(135)
RBM20	Colorectal cancer (CRC)	Not reported (SNP near RBM20)	Regulates T cell responses	SNP rs4918567 near RBM20 associated with clonality; eQTL for ADRA2A	Associated with T cell clonality/diversity	(136)
RBM47	Pancreatic cancer	High	Promotes progression & immune evasion	Binds PDIA6 3’-UTR → stability; alters metabolites	Weakens NK sensitivity; promotes evasion	(59)
RBMX	Osteosarcoma	High	Mediates immunosuppressive TME	KO → increases CD8+ T infiltration; upregulates H2-K1, downregulates THBS1	Enhances CD8+ T cytotoxicity; reshapes TME	(137)
RBMX	Various cancers (focus on liver)	Aberrant	Relevant for prognosis & immunotherapy	Knockdown impairs proliferation/migration/invasion in liver cancer; regulates immune	Enhances anti-tumor immunity	(138)

### 5.1 RNA mis-processing as a trigger of innate immune activation

RBPs have been shown to play a crucial role in regulating immunological responses in both inflammation and malignancy (29, 116). Current studies have underscored the dual ability of RBPs to either control pathological inflammation or be harnessed to activate antitumor immunity, depending on the cellular context (29, 116). In the lung, the Cold-shock RNA-binding protein RBM3 was revealed to be a natural suppressor of activated innate lymphoid cells (ILCs) (116). RBM3 is highly expressed in lung ILCs, where it is inducible by epithelial alarmins such as IL-33 and TSLP, making it a key regulator of type-2 inflammation (116). The conditional knockout of RBM3 results in exaggerated eosinophilic airway inflammation, with a concomitant increase in the production of type 2, as well as type 17, cytokines after exposure to allergen challenge (116). Bone marrow chimerism experiments confirm the ILC-intrinsic nature of the above-described phenotype, ruling out a secondary defect in the adaptive immune system (116). Transcriptomic studies have further implicated the upregulation of cysteinyl leukotriene signaling, including CysLT1R, in the above-described molecular defect, thus establishing a molecular pathway underlying the exacerbated inflammation in the absence of RBM3 (116). The genetic knockout of CysLT1R was sufficient to selectively attenuate the ILC defect, leading to a dislocation of ST2^+^/IL-17^+^/ST2α^+^ ILCs.

**Figure 4:**
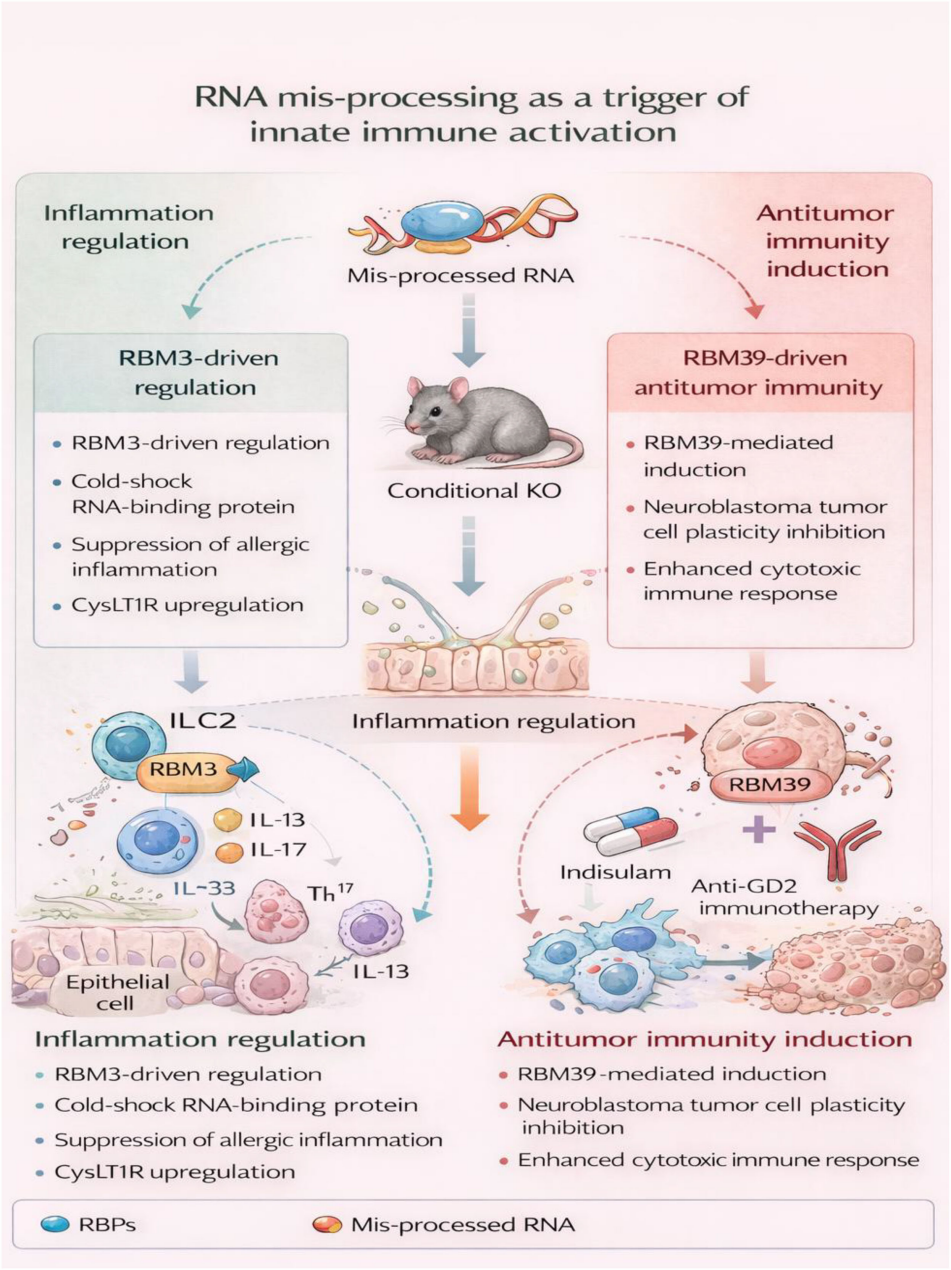
RNA mis-processing as a trigger of innate immune activation. RNA-binding proteins (RBPs) critically regulate innate immune responses by controlling RNA processing, thereby modulating inflammatory and antitumor programs in a context-dependent manner. In the lung, RBM3 acts as a suppressor of pathological type 2 and type 17 inflammation by restraining innate lymphoid cell activation, with its loss leading to exaggerated cytokine production via CysLT1R signaling. Conversely, pharmacological degradation of RBM39 disrupts tumor cell plasticity, reshapes the tumor microenvironment toward a pro-inflammatory state, and enhances natural killer cell–mediated cytotoxicity. These findings highlight RNA mis-processing as both a driver of inflammatory pathology and a therapeutic opportunity to activate innate antitumor immunity.

Conversely, a separate RBP, RBM39, maintains the plasticity of tumor cells during neuroblastoma by controlling the splicing of RNAs related to the adrenergic and mesenchymal phenotypes (29). The pharmacologically mediated degradation of RBM39 by the drug “indisulam” eliminates the plasticity of the tumor cells and induces a profound reshaping of the transcriptome and epigenome (29). Notably, RBM39 degradation restructures the tumor microenvironment into a pro-inflammatory niche and augments the cytotoxic effects of natural killer cells. Combination treatment using the drug “indisulam” and “anti-GD2 immunotherapy” causes sustained and complete regression of the high-risk neuroblastoma tumors irrespective of the status of the tumor cells (29). These observations collectively emphasize the context-dependent roles of RBPs: as natural inhibitors of inflammatory pathologies or as therapeutic agents that can tap the innate immune system’s power to target and eliminate cancer (**Figure 4**).

### 5.2 RBM-dependent suppression of adaptive anti-tumor immunity

RBM proteins, on the other hand, are believed to play a crucial role in modulating the tumor microenvironment (TME), specifically in evading adaptive anti-tumor immunity. RBM proteins modulate immunity evasion by affecting gene expression via mechanisms such as mRNA stability, translation efficiency, and epigenetic recruitment. However, these molecular changes should not be equated automatically with effective immune escape, because the net phenotype also depends on antigen presentation, cytotoxic lymphocyte function, and treatment context. In nasopharyngeal carcinoma (NPC), RBM3 mediates immunity resistance by increasing interleukin-10 (IL-10) secretion via the AKT/MEK1 signaling pathway, which attracts regulatory T cells (Tregs) and polarizes macrophages to M2 during an immunosuppressive tumor microenvironment that co-expresses PD-1/PD-L1 protein without changing programmed death-ligand 1 (PD-L1) expression levels (117). This RBM3 signaling mechanism generates a feedback mechanism where the immunoresistant TME also increases RBM3 expression in tumor cells to modulate evasion from CD8+ T cell-mediated cytotoxicity.

On the other hand, the tumor-suppressive role of RBM10 is seen in LUAD and PAAD, where its absence is associated with the augmentation of anti-tumor immune responses through the upregulation of human leukocyte antigen (HLA), tumor mutation burden (TMB), and the infiltration of CD8+ T lymphocytes, myeloid dendritic cells, macrophages, and neutrophils, accompanied by the augmentation of immune checkpoints such as PD-L1 and TIM-3 (37). In PAAD, RBM10 acts by suppressing immune evasion through the inhibition of PD-1 in natural killer (NK) lymphocytes by the downregulation of JAK/STAT signaling, thereby re-establishing NK lymphocytic cytotoxicity, whereas its absence leads to the activation of phosphorylated JAK1, JAK2, and STAT3, resulting in PD-1-induced NK lymphocytic exhaustion (120). Nonetheless, in pathologic N1-N2 EGFR-mutant LUAD, high RBM10 levels are associated with shorter overall survival and shorter progression-free survival (PFS) with EGFR-tyrosine kinase inhibitors (TKIs), suggesting a potential role in immune evasion, possibly through PD-L1 overexpression (119).

RBM12 mediates PD-L1-induced evasion of HCC by binding to JAK1 mRNA through its fourth RNA recognition motif (RRM) domain and recruiting eukaryotic initiation factor 4A2 (EIF4A2) through its second RRM domain, to promote JAK1 translation and activate JAK1/STAT1 signaling to induce PD-L1 expression (121) transcriptionally. Similarly, breast cancer-associated RBM15 regulates karyopherin alpha 2 (KPNA2) mRNA via N6-methyladenosine (m6A) modifications to promote proliferation, migration, invasion, and immune evasion by inhibiting CD8+ T cell proliferation and survival through apoptosis, and by concealing PD-L1 signaling (121). In LUAD, RBM15 is associated with immune infiltration and poor survival, as it highly enriches pathways such as cell cycle checkpoints/M phase, suggesting an indirect pathway that inhibits adaptive immunity (135).

RBM30 in HCC recruits the disruptor of telomeric silencing 1-like (DOT1L) protein to the STAT1 promoter, increasing histone H3 lysine 79 trimethylation (H3K79me3) and chromatin accessibility for the transcription factor STAT1, leading to the overexpression of PD-L1 and the exhaustion of T cells (9). RBM47 in gliomas and pancreatic cancer (PC) promotes immunosuppression; in gliomas, RBM47 is associated with high-grade tumors, shorter OS, and CD163+ macrophage accumulation, reflecting M2 polarization and the impaired function of effector T cells (122). In PC, RBM47 upregulates protein disulfide isomerase family A member 6 (PDIA6) by binding its 3’-untranslated region (UTR), stabilizing mRNA to enhance proliferation and NK cell evasion, with metabolomics revealing altered cellular metabolites that support this axis (59).

In osteosarcomas, as well as in cancers, RBMX functions as a mediator of CD8+ T cell suppression, downregulating histocompatibility 2, K1, and the K region (H2-K1), while also enhancing thrombospondin 1 (THBS1), reflecting decreased CD8+T cell migration/infiltration and cytotoxicity (137). Dysregulation of RBMX has been recognized in various cancers, suggesting a link to decreased survival, decreased cellular function after gene knockdown, and varying levels of immunotherapeutic efficacy, for example, immune infiltration (138). Germline variants in regions near RBM20 have also been identified as a factor for decreased T-cell receptor clonality in CRC, a condition that is also related to expression of alpha-2A adrenergic receptor (ADRA2A) in colon mucosa, indicating a relation to inherited genetic factors in the regulation of adaptive immunity (136). Although not a canonical RBM, the m6A reader IGF2BP2 in CRC stabilizes cell migration-inducing hyaluronidase (CEMIP) mRNA, promoting angiogenesis and metastasis via GRP78/PI3K-AKT activation in endothelial cells, indirectly suppressing adaptive immunity (123). These results emphasize the dual functions of RBM proteins, which regulate immune suppression by converging signaling pathways such as JAK/STAT signaling, mRNA stabilization, and epigenetic regulation to suppress CD8+ T cells and NK cells, but to induce Treg cells and M2 macrophage polarization. The dual functions of specific RBMs (e.g., RBM10) emphasize tumor specificity in therapeutic strategies.

**Figure 5:**
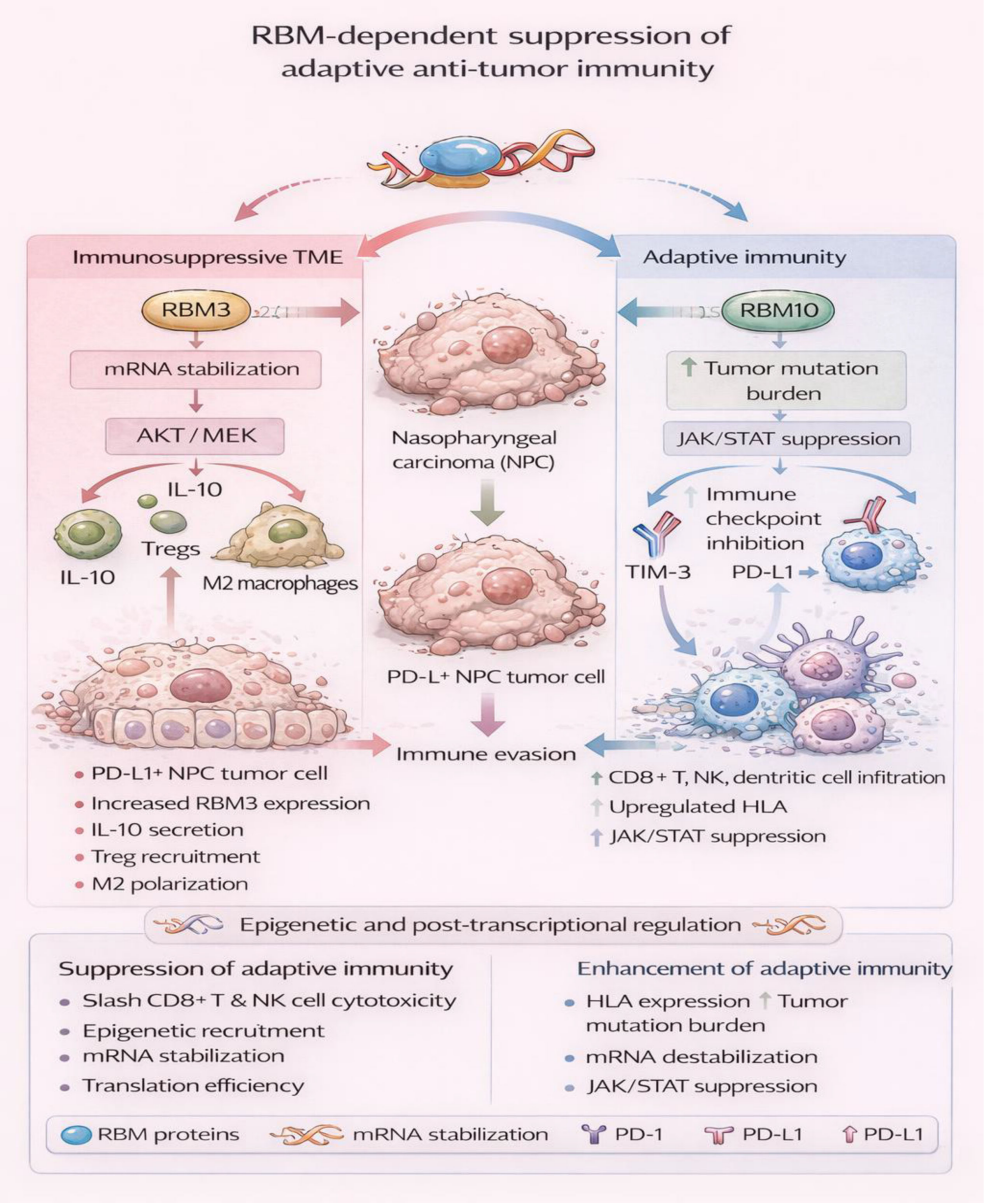
RBM-dependent suppression of adaptive anti-tumor immunity. RBM proteins modulate the tumor microenvironment by regulating post-transcriptional and epigenetic programs that shape adaptive immune responses. In nasopharyngeal carcinoma, RBM3 promotes immune evasion by enhancing IL-10 secretion through AKT/MEK signaling, leading to regulatory T cell recruitment, M2 macrophage polarization, and suppression of CD8^+^ T cell cytotoxicity. Conversely, loss or inhibition of RBM10 augments adaptive anti-tumor immunity by increasing tumor mutation burden, antigen presentation, and infiltration of cytotoxic lymphocytes through modulation of JAK/STAT and immune checkpoint pathways. Together, these context-dependent RBM-driven mechanisms highlight how RNA regulation governs immune suppression or activation and influences immunotherapy responsiveness.

Taken together, these studies support an immunoregulatory role for RBMs, but the strength of evidence varies across mechanisms. Findings such as PD-L1 induction, checkpoint enrichment, or macrophage polarization are hypothesis-generating when they are not accompanied by cytotoxicity assays, immune-cell depletion experiments, checkpoint-blockade response data, or clinical outcome correlations (37, 117, 119–123, 135–138). Conversely, the most persuasive immune conclusions come from models in which RBM perturbation changes NK-cell activity, CD8+ T-cell function, or treatment response directly. Future work should therefore distinguish pathway association from validated immune consequence more explicitly (**Figure 5**).

### 5.3 Remodeling of the tumor microenvironment via RBM-regulated cytokine and metabolic programs

RBM proteins drive TME remodeling by modulating cytokine circuits and metabolism. In the context of esophageal squamous cell carcinoma (ESCC), RBM15 induced the expression of procollagen-lysine, 2-oxoglutarate 5-dioxygenase 3 (PLOD3), thereby increasing CD4+ T-cell infiltration within the tumor and predicting a good prognosis, and formed a two-gene signature with PLOD3 to predict prognosis (124). In triple-negative breast cancer (TNBC), RBM15 increases paclitaxel resistance by increasing N6-methyladenosine (m6A) methylation of tumor necrosis factor superfamily member 9 (TNFSF9), which stimulates tumor-associated macrophage (TAM) polarization to M2 with high secretion of interleukin-10 and TGF-β and low secretion of IL-1β and tumor necrosis factor-α (TNF-α), to suppress anti-tumor immunity (125).

In colon adenocarcinoma (COAD), RBM15 maintains the stability of integrin beta-like 1 (ITGBL1) mRNA via m6A modification, which enhances M2 macrophage markers and EMT but suppresses CD8+ T cell proliferation, triggering their apoptosis to support the creation of an immunosuppressive TME (126). In a similar manner, in the case of Clear Cell Renal Cell Carcinoma (ccRCC), the RBM15 protein is induced by histone 3 hyperacetylation through EP300/CBP histone modifications within the gene’s promoter region; it maintains the stability of C-X-C motif chemokine ligand 11 (CXCL11) RNA through m6A modification to support the infiltration and polarization of macrophages to enhance proliferation, migration, invasion, and EMT (127). In PC, RBM15 influences m6A patterns to regulate macrophage phagocytosis; knockdown experiments suppress tumor growth but increase the macrophage infiltration and phagocytic activity against cancer cells (128).

In NSCLC, RBM15 enhances the m6A modification of CBR3 antisense RNA 1 (CBR3-AS1), which is recognized by IGF2BP3, stabilizing CBR3-AS1 to sponge microRNA-409-3p (miR-409-3p), leading to increased CXCL1 expression and recruitment of myeloid-derived suppressor cells (MDSCs) that repress T cell functions and induce radioresistance (129). In contrast, in CRC, RBM15 downregulation leads to increased expression of fumarate hydratase (FH), resulting in lowered levels of fumarate, a metabolic byproduct that represses anti-tumor immunity, resulting in increased infiltration and activation of CD8+ T cells and prolonged tumorigenesis (34). In another study that indirectly regulated RBM expression in CRC, exosomal long intergenic non-coding RNA 01615 (LINC01615) recruited RBMX to EZH2 mRNA and enhancer regions, leading to increased EZH2 expression and promoting M2 TAMs in tumorigenesis (130).

In prostate cancer (PCa), the overexpression in cancer cells and in TAMs is mediated by cytokine cross-talk, where M1 macrophages secrete the chemokine CXCL10, which binds to PCa cell surface receptor CXCR3, while M2 macrophages secrete IL-4 and IL-10, reciprocally upregulating RBM3, in turn stimulating overall translation of the gene for progression, as well as macrophage infiltration (131). In inflammatory conditions, such as rheumatoid arthritis (RA), genetic loss-of-function in RBM25 leads to exon skipping in pre-mRNA for ATP citrate lyase (ACLY), splicing towards the small ACLY S transcript that augments glycolysis, acetyl-CoA, histone acetylation via H3K9ac/H3K27ac, as well as activation of hypoxia-inducible factor 1-alpha (HIF1-alpha), concurring to upregulate pro-inflammatory mediators IL-1β, IL-6, and CXCL10 in macrophages (132). Intestinal injury models reveal that genetic loss of RBM47 affects proliferation, redox, and inflammatory signaling, contributing to upregulation of oxidative protection, Wnt signaling, and stem-cell protection against colitis, but promoting spontaneous polyposis and increased polyp burden in ApcMin/+ models (133).

In NSCLC, Pseudomonas putida KT2440 causes m6A modification of RBM47 mRNA by METTL3 and YTH N6-methyladenosine RNA-binding protein F1 (YTHDF1), which downregulates RBM47 to promote self-renewal of CSC and tumorigenesis by activating the Wnt signaling pathway, while stabilizing the PD-L1 mRNA by binding to the 3’-untranslated region (UTR) to inhibit proliferation and cytotoxicity of T cells (134). The findings emphasize the role of RBM proteins in playing a central role in the remodeling of the TME, which targets cytokine-mediated immune cell recruitment/programming (for example, M2-like TAM and MDSC), and metabolic shifts (for example, glycolytic activity and fumarate levels) to either suppress or enhance anti-tumor immunity, with context-dependent effects across tumor types.

**Figure 6:**
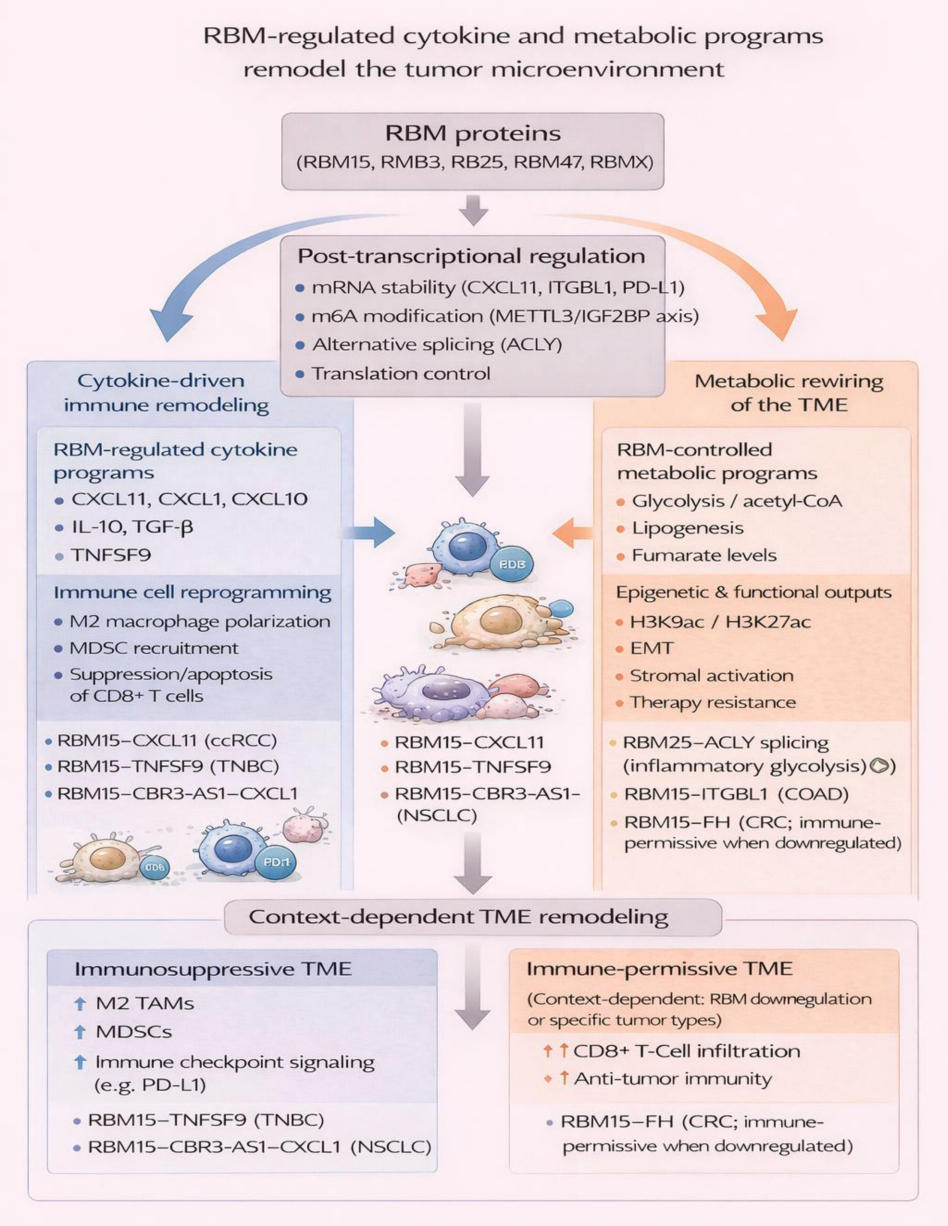
RBM-regulated cytokine and metabolic programs remodel the tumor microenvironment. RBM proteins coordinate post-transcriptional regulatory mechanisms, including mRNA stability, m6A modification, alternative splicing, and translation control, to shape the tumor microenvironment (TME). Through cytokine-driven programs, RBMs promote immune cell reprogramming by enhancing immunosuppressive macrophage and myeloid-derived suppressor cell infiltration while suppressing CD8^+^ T-cell activity. In parallel, RBM-controlled metabolic rewiring modulates glycolysis, lipogenesis, and epigenetic outputs, reinforcing immune suppression and therapy resistance in a context-dependent manner. Collectively, these RBM-mediated cytokine and metabolic networks drive divergent TME states, revealing cancer-type–specific vulnerabilities and therapeutic opportunities.

RBM-driven cytokine and metabolic remodeling provides a plausible route to immune exclusion, but the downstream effect is likely tumor specific. In some settings, RBM15-centered m6A programs enhance MDSC or macrophage recruitment and blunt T-cell function, whereas in others RBM loss appears to relieve metabolic immune suppression (34, 124–134). This diversity again argues against a single RBM-immunology model and supports biomarker-guided interpretation of each axis within its cellular and therapeutic context. (**Figure 6**).

## 6 RBM proteins in genome stability and therapeutic resistance: RNA-dependent regulation of DNA repair and adaptive responses

Genome stability is discussed here because RBM-controlled DNA repair and stress adaptation may shape both treatment response and tumor-immune behavior. By altering repair fidelity, damage persistence, or therapy-induced stress signaling, RBMs can influence not only survival after genotoxic therapy but also the conditions that determine antigenicity and inflammatory nucleic-acid sensing. The section therefore focuses on repair mechanisms with clear translational relevance rather than cataloging DNA damage biology in isolation (**Table 4**).

**Table 4:** RBM proteins in cancer therapy response: mechanisms of DNA repair, drug resistance, and prognostic significance.

RBM protein	Cancer type	Role	Key findings/mechanism	Prognostic implication	Ref
RBM6	Cancer (general, with focus on tumor suppressor activity)	Promotes HR repair; tumor suppressor	Regulates splicing-coupled nonstop-decay of Fe65/APBB1 → impairs HR; depletion sensitizes to cisplatin, ATM/PARP inhibition	RBM6-deficient tumors vulnerable to therapies	(139)
RBM39	HCC	Promotes BER	Stabilizes OGG1 mRNA → increases OGG1 → promotes BER; degradation + oxidative damage effective	Combined therapy for HCC	(140)
RBM39	MGMT-positive cancers (GBM, neuroendocrine)	Maintains MGMT	Depletion reduces MGMT → synergizes with O6-BG → enhances depletion	Dual targeting for MGMT+ tumors	(141)
RBM3	Epithelial ovarian cancer (EOC)	Good prognostic & cisplatin-sensitizing	Regulates DNA integrity genes (Chk1, Chk2, MCM3)	High RBM3 → prolonged survival; Chk1/Chk2/MCM3 → poor survival	(142)
RBM39	Breast cancer	Modulates splicing during stress	c-Jun interacts with RBM39 → prevents binding to pre-mRNA → alters splicing (COASY); contributes to cisplatin resistance	Not reported	(143)
RBM10	Lung adenocarcinoma (LUAD)	Regulates replication stress	Binds PRIM1 → recruits HDAC1 → H4K16 deacetylation/R-loop homeostasis → replication fork stability; RBM10-WEE1 synthetic lethality	Vulnerability to replication stress inducers	(144)
RBM3	Metastatic colorectal cancer (mCRC)	Good prognostic & oxaliplatin response	Not reported	High → prolonged survival, especially with oxaliplatin	(145)
RBM3	Muscle-invasive bladder cancer (MIBC)	Good prognostic & cisplatin-sensitizing	Regulates DNA damage response (Chk1, Chk2, MCM3)	High → prolonged survival & better cisplatin response	(146)
RBM5	Small cell lung cancer (SCLC)	Tumor suppressor & cisplatin-sensitizing	Regulates cell cycle & apoptosis	Reduces growth & increases cisplatin sensitivity	(55)
RBM10	Lung adenocarcinoma (LUAD)	Negative prognostic/predictive	Enriched in EGFR-mutant; co-occurs with L858R; higher in early stages	Mutations enrich in EGFR-mutant; concurrent TP53/KRAS → aggressive	(147)
RBM10	Non-small-cell lung cancer (NSCLC)	Negative prognostic/predictive	Not reported	Mutations → aggressive disease; concurrent ZFHX3/EGFR → stable, KRAS/TP53 → aggressive	(148)
RBM15	Laryngeal carcinoma	Promotes DDP resistance	Enhances KDM5B stability via IGF2BP3 → downregulates FER1L4 → upregulates GPX4; upregulates KCNQ1OT1 → inhibits ACSL4 → inhibits ferroptosis	Not reported	(149)
RBM15	Bladder cancer	Promotes cisplatin resistance	Drives H3K18la via LDHA → activates RBM15 → m6A IGFBP3 → nuclear IGFBP3/p-EGFR/p-DNA-PKcs → DNA repair	Not reported	(150)
RBM15	Lung adenocarcinoma (LUAD)	Promotes osimertinib resistance	Enhances m6A SPOCK1 mRNA → EMT via bypass activation	High → poor prognosis in EGFR-mutant	(151)
RBM15	Lung adenocarcinoma (LUAD)	Promotes progression & palbociclib sensitivity	m6A STIL activation → cyclin D1/CDK4 signaling	Not reported	(152)
RBM19	Prostate cancer	Promotes progression under docetaxel	Binds SNHG21 → stability → SNHG21/PIM1 → mitochondrial homeostasis	Not reported	(153)
RBMX	Hepatocellular carcinoma (HCC)	Contributes to progression & sorafenib resistance	Binds/stabilizes BLACAT1 → autophagy/CSC	Not reported	(154)
PDHB-AS	Cervical cancer	Suppresses progression & cisplatin resistance	Inhibits Wnt/β-catenin via PABPC1 recruitment → inhibits Wnt7b	Not reported	(155)
RBMX	T-cell lymphomas	Predicts chemotherapy response	Not reported	Low → better response & prognosis	(156)

### 6.1 RNA processing as a determinant of DNA repair pathway choice

RNA processing can influence DNA repair pathway choice at an upstream regulatory level. RBM6 promotes homologous recombination by sustaining correct processing of transcripts required for repair competence, and its loss increases sensitivity to cisplatin as well as ATM or PARP inhibition [ (41). By contrast, RBM39 supports base excision repair chiefly through stabilization of OGG1 mRNA rather than alternative splicing, illustrating that RBM-mediated genome protection can be transcript-specific and mechanistically distinct (140). RBM39 also contributes to maintenance of MGMT-dependent alkylation tolerance, linking post-transcriptional control directly to temozolomide resistance (141). These examples show that RBM proteins influence repair not only at the spliceosome level but also through RNA stability and damage-response thresholds.

A parallel but distinct mechanism arises in the regulation of base excision repair in response to oxidative damage, where RBM-driven glycosylase mRNA stabilization underpins the repair of oxidized bases and the survival of cancer cells (140). Here, the interaction between RBM and RNA increases transcript stability, with no effect on splicing variant usage, supporting the concept that RBM proteins employ distinct mechanisms to safeguard the genome (140). In addition, this aberration renders cells sensitive to oxidative damage, implying that the efficiency of the BER pathway can be controlled through RNA stability rather than enzymatic function (140). The third parallel mechanism is the maintenance of alkylation damage tolerance via RBM-driven stabilization of the MGMT protein, thereby directly impacting resistance to alkylator chemotherapy (141). Depletion of RBM39 destabilizes the MGMT protein and enhances the DNA damage responses and temozolomide-induced apoptosis, thereby supporting the concept that a regulatory threshold exists at the post-transcriptional level in chemoresistance (141).

Collectively, these studies place RBMs upstream of repair pathway choice, damage tolerance, and stress-adaptive survival. Their significance extends beyond chemotherapy sensitivity because the balance between faithful repair and persistent damage may also modify mutational load, cytosolic nucleic-acid signaling, and the pool of therapy-induced neoantigens. At present, however, these immune consequences remain much less directly tested than the repair phenotypes themselves (139–144). This gap should be acknowledged explicitly when linking genome maintenance to immuno-oncology.

### 6.2 RBMs in genome integrity under genotoxic stress

RBMs are unveiled as important genome integrity sentinels, especially during the response to genotoxic stress elicited by chemotherapeutic drugs such as cisplatin, where their roles in mediating the DNA damage response and replication accuracy are shown to impact cancer cell survival and the emergence of cisplatin resistance. In the context of epithelial ovarian cancer (EOC), RBM3 is shown to be associated with cellular functions that preserve DNA integrity, as ascertained by gene set enrichment analysis using a dataset of 267 patients (142). Its high expression is associated with better survival outcomes due to cells’ greater sensitivity to cisplatin treatment, mediated by modulation of genes involved in the DNA replication and repair pathway (142). Reduction of RBM3 expression in the ovarian cancer A2780 cell line induces checkpoint kinase 1 (Chk1), Chk2, and minichromosome maintenance complex component 3 (MCM3), important players in DNA damage checkpoints and replication initiation (142). Elevated expression of these proteins at both mRNA and protein levels in independent EOC cohorts (n=267 and n=154) predicts shorter survival, with phosphorylated Chk2 serving as an adverse prognostic marker, indicating that RBM3 suppresses compensatory activation of DNA integrity pathways that could otherwise confer resistance to genotoxic insults (142).

Upon genotoxic stress, as induced by cisplatin in breast cancer cells, the transcription factor c-Jun and RBM39 interact to reprogram pre-mRNA splicing, thereby modulating genome-wide alternative splicing events that contribute to drug resistance (143). The protein-protein interaction inhibits RBM39 binding to its pre-mRNA targets, thereby producing defective isoforms, as evidenced by the short isoform of coenzyme A synthase (COASY) that lacks exons 4 and 5 (143). The silencing of this short COASY isoform inhibits mitochondrial function and reduces sensitivity to cisplatin, thereby demonstrating how RBM39 inactivation impacts genome stability through metabolic support (143). Through its inhibitory mechanism involving c-Jun, RBM39 activation orchestrates a partial overlap with a mechanism involving alternative splicing events induced by cisplatin, contributing to cell adaptations that maintain genome integrity at the expense of increased survival and resistance (143).

LUAD, with frequent RBM10 mutations (9-25%) resulting in loss of function and consequent hyper-tumorigenesis, is particularly sensitive to DNA replication stress response mechanisms, as shown in CRISPR-Cas9-based synthetic lethality screens targeting about 60 protein interactors, including the WEE1 kinase (144). Although RBM10-null cells in LUAD are highly sensitive to WEE1 inhibition in both *in vitro* and *in vivo* settings, this is due to its splicing-independent role in regulating the progression of replication forks (144). In this regard, RBM10 is recruited to active replication forks through its binding to the DNA primase subunit 1 (PRIM1), which is responsible for the synthesis of Okazaki RNA primers, where it anchors histone deacetylase 1 (HDAC1) in the process of histone H4 lysine 16 (H4K16) histone modification to maintain R-loop stability through histone H4K16 acetylation modification with the subsequent absence of its function in RBM10 mutation increasing replication stress susceptibility to WEE1-dependent checkpoint activation for survival in genotoxically stressed cells (144).

Taken together, these findings establish the RBMs as complex regulators that coordinate splicing-dependent versus -independent responses to genotoxic stress to preserve genome integrity, through RBM3’s modulation of DNA damage checkpoints to potentiate cisplatin responses, the c-Jun inhibition of RBM39 to repurpose splicing to resist, to RBM10’s integration of epigenetic marks to protect from stress-induced genome instability. These complex interactions not only help explain the clinical significance of RBM deregulation in EOC versus LUAD, with high Chk1/Chk2/MCM3/RBM10 loss predicting poor prognosis, but also underscore exploitable vulnerabilities to therapeutically abolish integrity-preserving pathways in genotoxin-treated cancers through synthetic lethal interactions, such as RBM10-WEE1.

### 6.3 Adaptive RNA programs underlying resistance to chemotherapy and radiotherapy

The involvement of RBPs and their associated ncRNAs encompasses adaptive programs that regulate responses to genotoxic therapies, including platinum drugs used in chemotherapeutic treatment, with repurposing strategies to evade cell survival through splicing, stability, and epigenetic alterations. In metastatic colorectal cancer (mCRC), high nuclear and cytoplasmic expression of RBM3 independently predicts OS and PFS with first-line oxaliplatin versus irinotecan-based treatment, respectively, indicating synergistic oxaliplatin sensitivity via mechanisms that promote DNA damage and activate repair pathways (145). In muscle-invasive bladder cancer (MIBC), high tumor protein expression of RBM3 by transurethral biopsy correlates with faster TRR, CSS, OS hazards among patients treated with first-line cisplatin-based NAC therapy, where silencing RBM3 in high-grade T24 cells renders cisplatin/S1 sensitivities to treatment via inhibiting cell cycle G1/S progression initiation with singular G1 accumulation with altered levels for CDC2/CDK4/and CDK2 mRNAs relative to Cln1/2 and CDC2 mRNAs (146), thereby indicating adaptation to an unloaded cell cycle transcriptome regulated by RBM3 to evade cell cycle arrest due to chemotherapeutic treatment.

In small cell lung cancer (SCLC), the restoration of RBM5 expression in GLC20 cells, which have a homozygous deletion of the RBM5 locus, retards growth and increases cisplatin sensitivity through direct pathway involvement of cell cycle, apoptosis, angiogenesis, and cell adhesion, as validated by transcriptome analysis and functional studies reflecting diminished proliferation rates and increased susceptibility to drugs as shown (55). In the broader context of LUAD, mutations in the RBM10 gene, particularly in the context of EGFR mutations, are linked to early-stage tumors in the absence of EGFR mutations but to advanced-stage tumors in the presence of mutations in the latter, wherein “loss-of-function” (LOF) mutations are associated with increased rates of PD-L1 expression, Tumor Mutational Burden, and median PFS in response to immunotherapy using the PD-1 inhibitor (147). In the context of NSCLC, the mutation of the “tumor suppressor” RBM10 leads to “aggressive progression” of the disease associated with a median PFS of 6.7 vs 13.9 months for the “wildtype,” and whose severity is further “unmasked” but “mitigated” by the co-mutations, indicating RBM10’s role in stabilizing genomic integrity against chemotherapy (148).

RBM15 exemplifies m6A-dependent adaptive resistance, in laryngeal carcinoma (LC) where it upregulates lysine-specific demethylase 5B (KDM5B) mRNA stability via insulin-like growth factor 2 mRNA-binding protein 3 (IGF2BP3), leading to downregulation of lncRNA Fer-1 like family member 4 (FER1L4) and upregulation of glutathione peroxidase 4 (GPX4), while KDM5B elevates lncRNA KCNQ1 overlapping transcript 1 (KCNQ1OT1) to inhibit Acyl-CoA synthetase long-chain family member 4 (ACSL4), collectively suppressing ferroptosis by reducing iron, reactive oxygen species (ROS), and malondialdehyde while increasing glutathione, thereby promoting cisplatin (DDP) resistance (149). In the case of bladder cancer (BCa), the release of lactate from lactate dehydrogenase A (LDHA) due to hypoxia triggers histone 3 lysine 18 lactylation (H3K18la) of the RBM15 gene via KAT2B/KAT8/MIZ1, activating RBM15 transcription; this subsequently m6A-modifies the IGFBP3 mRNA for stability, facilitating the translocation of the IGFBP3/EGFR/DNA-PKcs complex from the nucleus for DNA repair and protection against cisplatin-induced damage (150). In the context of LUAD, the overexpression of RBM15 is a predictor of poor clinical outcomes in EGFR mutants and contributes to the resistance of EGFR mutants to the medication osimertinib via the m6A modification of cwcv- and kazal-like domains proteoglycan 1 mRNA for the subsequent induction of the EMT bypass pathway in SPOCK1 (151). The transcription factor RBM15 subsequently activates SCL-interrupting locus (STIL) expression via the m6 to upregulate cyclin D1/CDK4 signaling, enhancing proliferation, migration, and G2/M progression, though sensitizing to palbociclib by attenuating CDK4/6 inhibition (152).

Moreover, RBM19 in PCa interacts with the lncRNA small nucleolar RNA host gene 21 (SNHG21), protecting the proviral integration site for Moloney murine leukemia virus 1 (PIM1) from ubiquitin-mediated degradation, as determined by RNAi screens and *in vivo* and *in vitro* analyses (153). Additionally, the RBMX in HCC interacts with the lncRNA bladder cancer-associated transcript 1 (BLACAT1), thereby increasing autophagy, stemness, and sorafenib resistance, as demonstrated by RNAi analysis and *in vivo*/*in vitro* assays (154). On the contrary, in cervical cancer, the lncRNA pyruvate dehydrogenase E1 subunit beta antisense (PDHB-AS) interacts with RBMX to suppress Wnt7b and the Wnt/β-catenin subcellular localization through the binding of Dickkopf-1 (DKK1) with miR-582-5p. Simultaneously interacting with poly(A) binding protein cytoplasmic 1 (PABPC1) inhibits proliferation, invasion, and cisplatin resistance (155). In T-cell non-Hodgkin’s lymphomas (T-NHL), decreased RBMX expression predicts favorable anthracycline responsiveness, OS, and progression-free survival, and indicates an oncogenic role for RBMX in chemotherapy resistance by stabilizing oncogenic transcripts (156).

**Figure 7:**
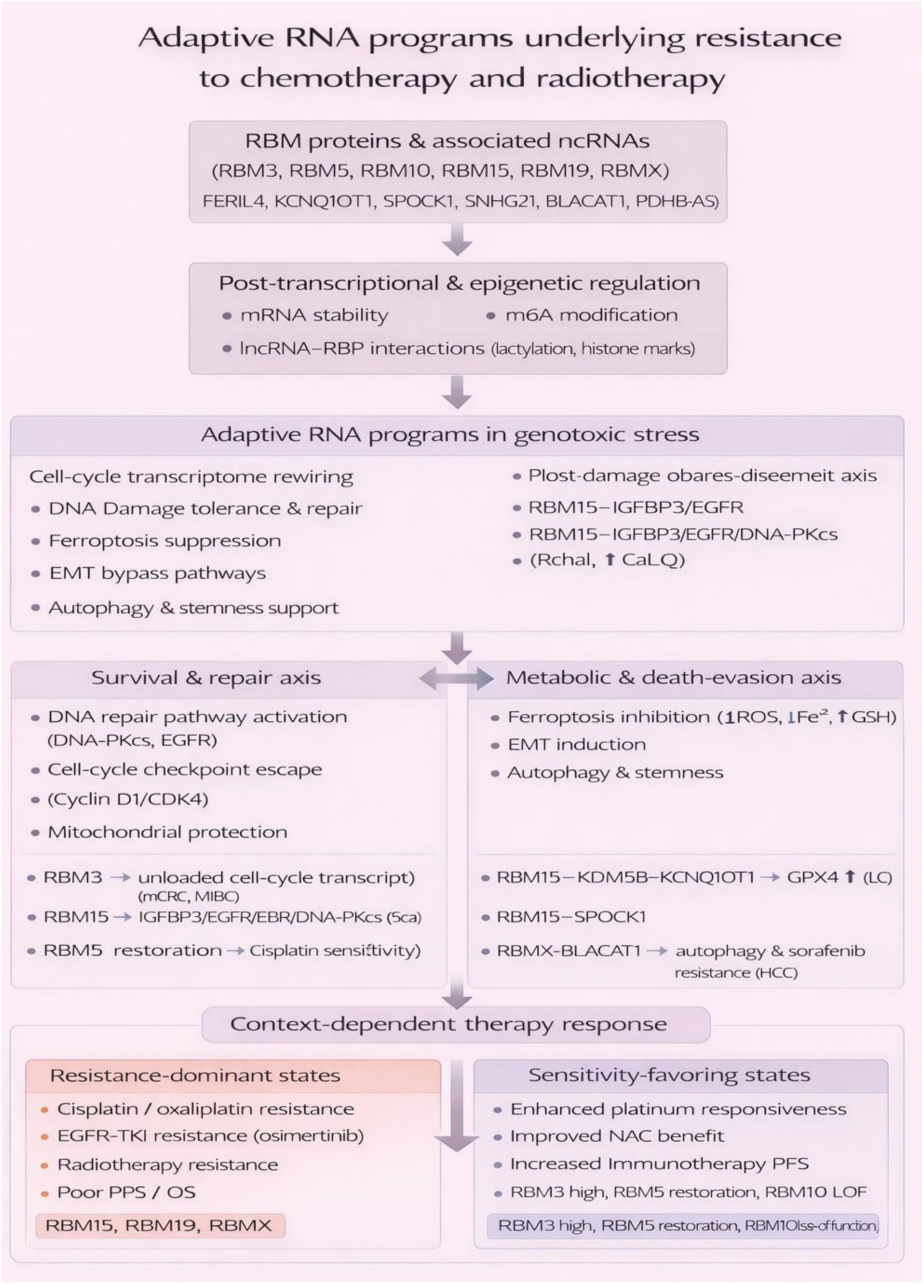
Adaptive RNA programs underlying resistance to chemotherapy and radiotherapy. RNA-binding motif (RBM) proteins and associated noncoding RNAs orchestrate post-transcriptional and epigenetic programs that enable tumor adaptation to genotoxic stress. Through modulation of RNA stability, alternative splicing, m6A-dependent regulation, and lncRNA–RBP interactions, these programs rewire cell-cycle control, DNA damage repair, ferroptosis, EMT, and autophagy pathways. The resulting context-dependent RNA states drive either therapeutic resistance or sensitivity across cancer types, shaping clinical responses to chemotherapy.

These adaptive RNA programs converge on ferroptosis suppression, DNA repair, EMT, and stemness, but their translational relevance will depend partly on whether they also alter immune visibility after treatment. At present, evidence is strongest for therapy resistance itself, whereas links to antigenicity, interferon signaling, or checkpoint responsiveness remain suggestive rather than established in most tumor types (145–156). This distinction should be kept explicit when interpreting RBM-driven resistance phenotypes (**Figure 7**).

## 7 Pharmacological targeting of RBM proteins: disruption of oncogenic RNA networks and overcoming therapeutic resistance

Therapeutic targeting of RBMs can be grouped into three strategies: disruption of oncogenic RNA programs, targeted degradation of RBM proteins, and interception of acquired resistance pathways. The discussion below emphasizes not only efficacy signals but also selectivity, resistance mechanisms, and translational limitations (**Table 5**).

**Table 5:** Pharmacological degradation of RBM proteins: molecular glue mechanisms, therapeutic efficacy, and acquired resistance in cancer.

RBM protein	Cancer type	Role in cancer	Key findings/mechanism	Ref
RBM10	Neuroblastoma	Promotes progression	Interacts with RORB in condensates → represses RORB transactivation; peptide blocks interaction → inhibits lysosomal biogenesis/NF-κB	(157)
RBM39	High-grade serous ovarian carcinoma	Degradation inhibits progression	Induces splicing defects in DNA repair genes → increases sensitivity to cisplatin/PARP inhibitors	(158)
RBM39	Neuroblastoma	Degradation exerts anti-cancer	Alectinib inhibits SRPK1 → enhances degradation → synergistic with indisulam → RNA splicing defects/DNA damage	(159)
RBM39	General (hematopoietic/lymphoid)	Degradation causes splicing defects	Recruits RBM39 to CUL4-DCAF15 → ubiquitination/degradation → RNA splicing defects	(160)
RBM39/RBM23	General	Degradation causes splicing defects	Sulfonamides recruit to CRL4-DCAF15; RBM39 via RRM2 → ubiquitinated on N-terminus	(161)
RBM39	Neuroblastoma (high-risk)	Degradation therapeutic	Degrades RBM39 → disrupts metabolism/mitochondria → complete regression	(162)
RBM39	Neuroblastoma	Vulnerability	Indisulam degrades RBM39 → splicing anomalies/cell death; high DCAF15 → sensitivity	(163)
RBM39	T-cell acute lymphoblastic leukemia	Degradation anti-cancer	Degrades RBM39 via DCAF15 → splicing anomalies/apoptosis; THOC1 as effector	(32)
RBM39	Acute megakaryoblastic leukemia	Degradation inhibits	Degrades RBM39 → splicing of ZMYND8	(164)
RBM39	Non-small cell lung cancer	Enhances resistance to Indisulam	PRMT6 methylates RBM39 at R92 → inhibits degradation → promotes splicing/proto-oncogenes	(165)
RBM39	Head and neck squamous cell carcinoma	Interaction confers resistance	YAP/TAZ delays degradation → restores integrin/collagen/FAK → R-loop/DNA damage	(166)
RBM39	Gastric cancer	Stabilized by USP39 promotes progression	USP39 deubiquitinates RBM39 (K48) → stabilizes → promotes growth/migration/invasion	(167)

### 7.1 Pharmacological targeting of RBM-driven RNA programs and therapeutic synergies

Pharmacological interventions targeting RBM proteins aim to leverage their roles in RNA splicing, transcription regulation, and liquid-liquid phase separation to interfere with oncogenic RNA expression, often used in a synergistic manner with conventional therapies to overcome resistance. In neuroblastoma, a short peptide selectively inhibiting interaction within liquid condensates between RBM10 and RAR-related orphan receptor B (RORB) reestablishes the transcriptional activity of RORB, which increases expression levels for nuclear receptor subfamily 1 group D member 1 (NR1D1) and RIO kinase 3 (RIOK3) in a circadian rhythm-dependent manner, resulting in down-regulation of nuclear factor kappa B (NF-κB), consequent to derepression of folliculin (FLCN) and folliculin interacting protein 1 (FNIP1), impairment in lysosomal biogenesis, growth, invasiveness, and metastasis, as well as suppressed tumorigenicity (157).

In high-grade serous ovarian carcinoma (HGSC), the molecular glue agent indisulam (E7070) causes polyubiquitination and degradation of RBM39 by targeting it to DCAF15 E3 ubiquitin ligase, leading to splice defects in RNA molecules crucial for DNA damage repair genes, thereby sensitizing to cisplatin and poly-ADP ribose polymerase (PARP) inhibitors, with combined therapy showing efficacy also in PARP inhibitor-resistant xenograft models of HGSC (158). In high-risk neuroblastoma, the degradation of RBM39 by the molecular glue agent indisulam synergizes with alectinib, an ALK inhibitor targeting Ser/Arg protein kinase 1 (SRPK1), to exacerbate RNA splice defects in DNA damage response/R-loop resolution pathways, resulting in R-loop accumulation, augmented DNA damage, apoptosis, cell cycle arrest, and complete tumor regression in Th-MYCN/ALKF1174L models, with significant survival benefit over monotherapies (159). These strategies highlight therapeutic targeting strategies for harnessing anti-tumor activity through degradative, interaction-blocking, or rational combination inhibition, focusing on RNA programs regulated by RBMs and emphasizing pathway alterations driven by splicing defects.

These therapeutic concepts are promising but should be discussed with balanced caution. Spliceosome-directed interventions can produce mechanism-based toxicity in normal proliferative tissues because RNA-processing dependencies are not tumor exclusive (157–159). Their activity is also unlikely to be uniform across cancers, since efficacy depends on factors such as DCAF15 abundance, RBM conformation, and lineage-specific splicing liabilities. In addition, most evidence remains preclinical, so claims about broad clinical applicability should be tempered until biomarker-defined trials confirm benefit.

### 7.2 Targeted protein degradation as a strategy to collapse splicing-dependent oncogenic states

Targeted degradation of RBM proteins, particularly through molecular glues like indisulam, disrupts oncogenic splicing programs by inducing proteasomal turnover via E3 ubiquitin ligases, leading to widespread pre-mRNA splicing aberrations that collapse cancer cell states reliant on precise RNA processing. Indisulam acts as a molecular glue to recruit RBM39 to the CUL4-DCAF15 E3 ubiquitin ligase, promoting RBM39 polyubiquitination and degradation, which triggers aberrant pre-mRNA splicing and cytotoxicity selectively in hematopoietic and lymphoid cancer cell lines, where sensitivity correlates with DCAF15 expression levels; mutations in RBM39’s RNA recognition motif 2 (RRM2) prevent recruitment and confer resistance, as do clinically tested analogs tasisulam and chloroquinoxaline sulfonamide, defining a class of splicing inhibitor sulfonamides (SPLAMs) (160). Similarly, indisulam and related sulfonamides degrade both RBM39 and its paralog RBM23 via CRL4-DCAF15, with RBM39’s RRM2 domain mediating recruitment to DCAF15’s Q232 and D475 residues; this induces over 3,000 gene expression changes, intron retention, and exon skipping, all attributable to RBM39 loss rather than RBM23, which is ubiquitinated on its N-terminus (161).

In high-risk neuroblastoma (NB), indisulam induces rapid DCAF15-dependent RBM39 degradation, accumulating splicing errors and inhibiting growth through integrative RNA-seq and proteomics, revealing disruptions in cell cycle and metabolism, including metabolome perturbations and mitochondrial dysfunction, with complete tumor regression in xenograft and Th-MYCN transgenic models without relapse (162). The mechanism of action of indisulam against NB is genome-wide splicing errors caused by degradation of the target protein RBM39, leading to strong responses in high-risk models, such as xenografts, without notable toxicities, making the concept of spliceosome targeting a vulnerability of MYC-amplified tumors (163). In the case of T-cell acute lymphoblastic leukemia (T-ALL), the mechanism of action of indisulam is the promotion of the degradation of the target protein RBM39, which regulates the mis-splicing of pre-mRNAs, reducing cell growth, increasing apoptosis, and arresting the cell cycle through the reduction of the downstream target proteins, such as the THO complex 1 subunit (THOC1), with a knockout of the DCAF15 protein that abrogates the action and demonstrates the dependence on RBM39 (32). In the case of acute megakaryoblastic leukemia (AMKL), the mechanism of action of indisulam is the promotion of the degradation of the target protein RBM39, which modulates the alternative splicing of the ZMYND8 gene, which regulates the growth of the acute megakaryoblastic cells, reducing the amount of the tumor and the survival of the mice in the model of the disease, depending on the expression of the DCAF15 protein, which, if knocked out, prevents the decrease of the stability of the target protein and the splicing of ZMYND8 (164). These mechanisms highlight targeted RBM degradation as a potent strategy to dismantle splicing-dependent oncogenic dependencies, leveraging ubiquitin ligase recruitment to induce catastrophic RNA processing errors across hematologic and pediatric malignancies.

**Figure 8:**
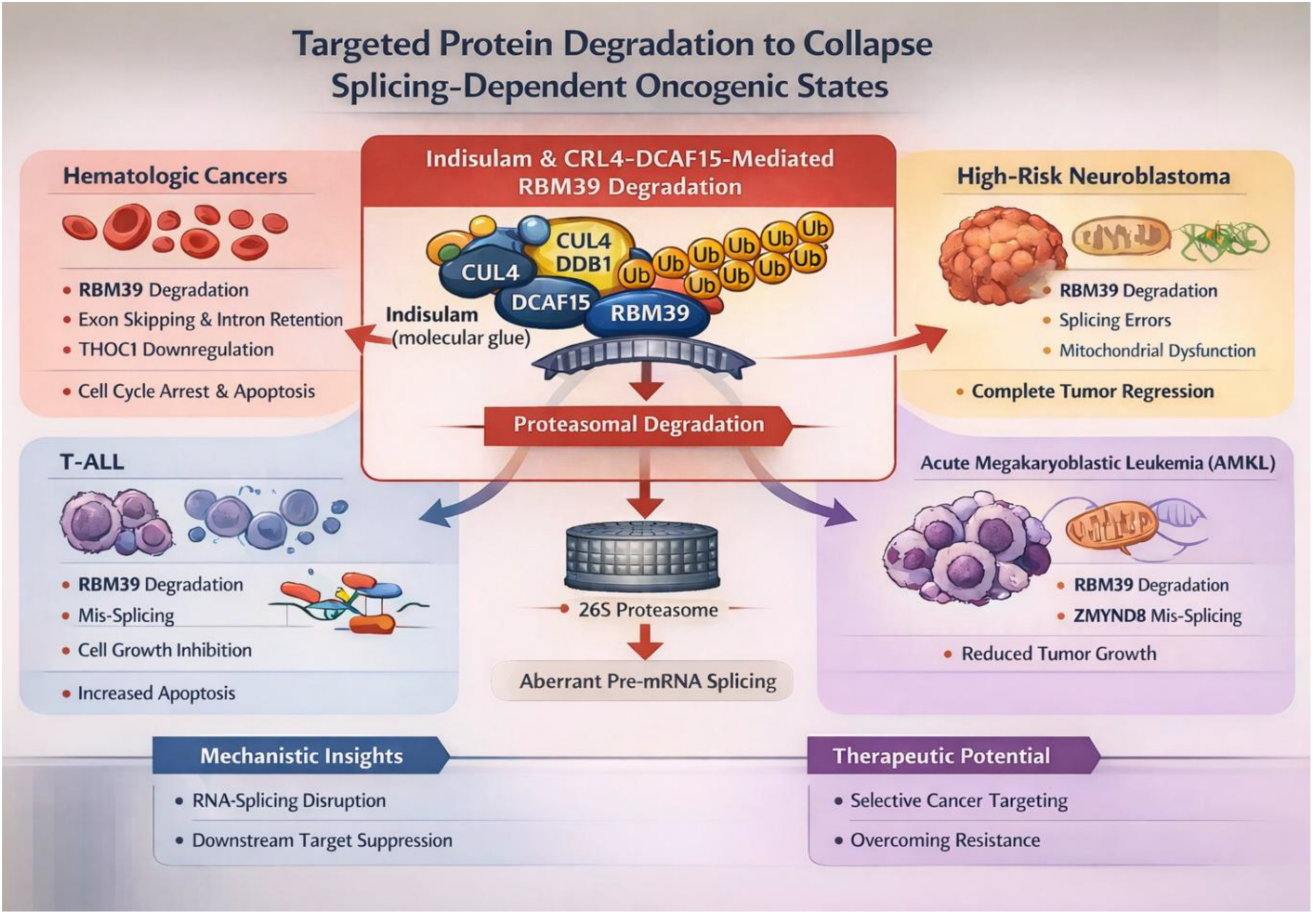
Targeted degradation of RBM39 collapses splicing-dependent oncogenic states. Indisulam functions as a molecular glue to recruit the splicing factor RBM39 to the CRL4–DCAF15 E3 ubiquitin ligase, triggering RBM39 polyubiquitination and proteasomal degradation. Loss of RBM39 induces widespread pre-mRNA splicing defects, including exon skipping and intron retention, leading to disruption of cell-cycle, metabolic, and RNA-processing programs. This mechanism selectively dismantles splicing-dependent vulnerabilities in hematologic malignancies and high-risk pediatric cancers, resulting in growth arrest, apoptosis, and tumor regression.

Targeted RBM39 degradation offers a compelling way to collapse splicing-dependent tumor states, particularly in malignancies with strong DCAF15 expression or pronounced spliceosomal vulnerability (160–164). Even so, this strategy should not be framed as universally selective. Sensitivity varies markedly across lineages, and resistance mutations or compensatory RNA-processing programs can blunt response. The main translational challenge is therefore not proof of mechanism, which is strong, but identification of tumors in which therapeutic window and dependency are both sufficient for durable benefit (**Figure 8**).

### 7.3 Mechanisms of resistance to RBM-targeting therapies

Resistance to RBM-targeting therapies most often reflects restoration of RBM39 stability or impaired degrader engagement. In NSCLC, PRMT6-mediated methylation of RBM39 at R92 blocks efficient recruitment to the CUL4-DCAF15 ligase complex and preserves oncogenic splicing programs (165). As a result, cell-cycle and anti-apoptotic transcripts remain supported despite indisulam exposure. Pharmacologic PRMT6 inhibition or R92A substitution restores RBM39 degradation and re-sensitizes cells, identifying methylation-dependent target shielding as a tractable resistance mechanism (165).

In head neck squamous cell carcinomas (HNSCCs), resistance is contributed to by an interaction between RBM39 and Hippo pathway target proteins Yes-associated protein/transcriptional coactivator with PDZ-binding motif (YAP/TAZ) (166). Hyperactivation of YAP/TAZ, commonly as a result of pathway misregulation within solid tumors, binds to RBM39, postponing its degradation through indisulam. The bindings happen in the nuclei, where YAP/TAZ protects RBM39 from E3 ligases to sustain its splicing function. This causes persistent regulation of pre-mRNA processing for integrins (ITGA5) and collagens (COL1A1), thereby activating Focal Adhesion Kinase (FAK) signaling (166). P-FAK phosphorylates downstream proteins, such as AKT/ERK, to initiate cell survival, migration, and resistance to apoptosis. Subsequently, stabilization of splicing for cell cycle regulators (CCND1) and DNA repair proteins (BRCA1) also occurs through YAP/TAZ-RBM39 to counteract splicing aberrations induced by indisulam treatment, thereby mitigating genomic instability. *In vitro* and *in vivo* models establish faster degradation via YAP/TAZ knockdown to reactivate resistance by inhibiting FAK pathways, due to splicing errors that prevent cell death (166).

USP39 promotes gastric cancer resistance by deubiquitinating RBM39. The protein USP39 colocalizes with RBM39 in the nucleus and hydrolyzes K48-linked polyubiquitin chains conjugated by E3 ligase to prevent proteasomal degradation and promote RBM39 stability (167). Colocalization enhances RBM39 splicing activity, thereby elevating the expression of oncogenes that promote proliferation (e.g., MYC targets) and invasiveness (e.g., MMPs). The role of RBM39 in promoting colonization, migration, and invasion is further reinforced by USP39 overexpression and reduced by USP39 knockdown. The role of USP39 involves its cysteine protease activity at the catalytic site (Cys-205), which is critical and cannot be inhibited by mutations that deplete deubiquitinating activity (167). RBM39-overexpressing plasmids can rescue the knockdown phenotypes induced by targeting RBM. Such dependency represents crucial adaptive mechanisms promoted through multiple pathways associated with RBM-targeted therapies.

**Figure 9:**
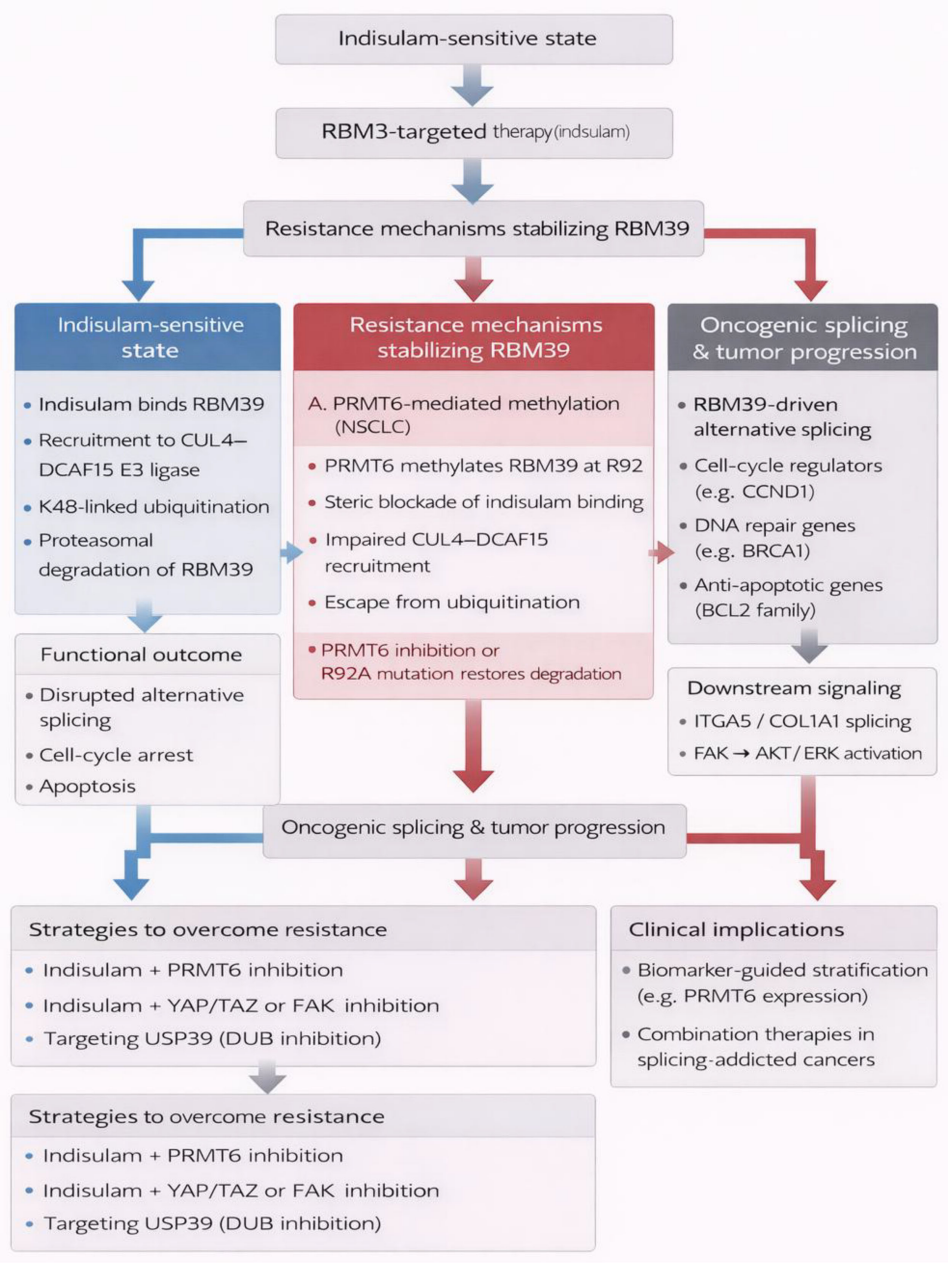
The molecular glue indisulam normally induces RBM39 degradation via the CUL4–DCAF15 E3 ligase, leading to splicing disruption and cell death. Resistance arises through RBM39-stabilizing mechanisms such as PRMT6-mediated methylation, YAP/TAZ interaction, or USP39-mediated deubiquitination. Combined therapeutic strategies—including PRMT6, YAP/TAZ, FAK, or DUB inhibition, can restore RBM39 degradation and sensitivity. Biomarker-guided combinations may improve outcomes in splicing-addicted cancers. All the figures are conceptual schematic synthesized from cited studies; no single experimental cohort or sample size applies.

Overall, resistance to RBM-targeted therapy is heterogeneous and likely multifactorial. PRMT6-dependent methylation, YAP/TAZ-mediated protection, and USP39-dependent deubiquitination illustrate three distinct escape routes: impaired degrader binding, target shielding, and restored protein stability (165–167). These data support rational combinations, but they also underscore that durable benefit will require predictive biomarkers, pharmacodynamic monitoring, and careful management of toxicity from broader spliceosomal perturbation. (**Figure 9**).

## 8 Conclusions

RBM proteins emerge as integrators of four interconnected regulatory layers: isoform selection, m6A-coupled RNA fate control, stress-adaptive genome maintenance, and immune microenvironment remodeling. Across tumor types, these layers converge on a limited set of outputs, namely proliferative signaling, plasticity, immune suppression, and therapeutic resistance. Yet RBM function is not inherently oncogenic or tumor suppressive; it depends on lineage-specific transcriptomes, partner proteins, chromatin state, and selective pressures from metabolism and immunity. Current evidence is strongest for mechanistic roles in RNA processing and treatment response, while direct links to clinical immune outcomes remain less mature. This distinction is important for translating RBM biology into biomarker strategies and therapeutic intervention.

Future directions should focus on high-resolution RBM interactome mapping using patient-derived organoids and single-cell multi-omics to delineate isoform-specific vulnerabilities. Developing selective degraders or ASOs targeting RBM–lncRNA axes could enable precision interventions, while integrating RBM signatures as biomarkers may stratify patients for combination regimens with checkpoint inhibitors or PARP inhibitors. Validation in diverse cohorts will be crucial to mitigate off-target effects and address tumor heterogeneity. Ultimately, decoding RBM orchestration promises to unlock novel immuno-oncologic paradigms, transforming intractable cancers into therapeutically tractable entities and broadening the horizon of curative strategies.
